# Measurement of the top quark mass using a profile likelihood approach with the lepton + jets final states in proton–proton collisions at $\sqrt{s}=13\,\text {Te}\hspace{-.08em}\text {V}$

**DOI:** 10.1140/epjc/s10052-023-12050-4

**Published:** 2023-10-25

**Authors:** A. Tumasyan, W. Adam, J. W. Andrejkovic, T. Bergauer, S. Chatterjee, K. Damanakis, M. Dragicevic, A. Escalante Del Valle, P. S. Hussain, M. Jeitler, N. Krammer, L. Lechner, D. Liko, I. Mikulec, P. Paulitsch, F. M. Pitters, J. Schieck, R. Schöfbeck, D. Schwarz, M. Sonawane, S. Templ, W. Waltenberger, C.-E. Wulz, M. R. Darwish, T. Janssen, T. Kello, H. Rejeb Sfar, P. Van Mechelen, E. S. Bols, J. D’Hondt, A. De Moor, M. Delcourt, H. El Faham, S. Lowette, S. Moortgat, A. Morton, D. Müller, A. R. Sahasransu, S. Tavernier, W. Van Doninck, D. Vannerom, B. Clerbaux, G. De Lentdecker, L. Favart, D. Hohov, J. Jaramillo, K. Lee, M. Mahdavikhorrami, I. Makarenko, A. Malara, S. Paredes, L. Pétré, N. Postiau, L. Thomas, M. Vanden Bemden, C. Vander Velde, P. Vanlaer, D. Dobur, J. Knolle, L. Lambrecht, G. Mestdach, C. Rendón, A. Samalan, K. Skovpen, M. Tytgat, N. Van Den Bossche, B. Vermassen, L. Wezenbeek, A. Benecke, G. Bruno, F. Bury, C. Caputo, P. David, C. Delaere, I. S. Donertas, A. Giammanco, K. Jaffel, Sa. Jain, V. Lemaitre, K. Mondal, A. Taliercio, T. T. Tran, P. Vischia, S. Wertz, G. A. Alves, E. Coelho, C. Hensel, A. Moraes, P. Rebello Teles, W. L. Aldá Júnior, M. Alves Gallo Pereira, M. Barroso Ferreira Filho, H. Brandao Malbouisson, W. Carvalho, J. Chinellato, E. M. Da Costa, G. G. Da Silveira, D. De Jesus Damiao, V. Dos Santos Sousa, S. Fonseca De Souza, J. Martins, C. Mora Herrera, K. Mota Amarilo, L. Mundim, H. Nogima, A. Santoro, S. M. Silva Do Amaral, A. Sznajder, M. Thiel, A. Vilela Pereira, C. A. Bernardes, L. Calligaris, T. R. Fernandez Perez Tomei, E. M. Gregores, P. G. Mercadante, S. F. Novaes, Sandra S. Padula, A. Aleksandrov, G. Antchev, R. Hadjiiska, P. Iaydjiev, M. Misheva, M. Rodozov, M. Shopova, G. Sultanov, A. Dimitrov, T. Ivanov, L. Litov, B. Pavlov, P. Petkov, A. Petrov, E. Shumka, S. Thakur, T. Cheng, T. Javaid, M. Mittal, L. Yuan, M. Ahmad, G. Bauer, Z. Hu, S. Lezki, K. Yi, G. M. Chen, H. S. Chen, M. Chen, F. Iemmi, C. H. Jiang, A. Kapoor, H. Liao, Z.-A. Liu, V. Milosevic, F. Monti, R. Sharma, J. Tao, J. Thomas-Wilsker, J. Wang, H. Zhang, J. Zhao, A. Agapitos, Y. An, Y. Ban, A. Levin, C. Li, Q. Li, X. Lyu, Y. Mao, S. J. Qian, X. Sun, D. Wang, J. Xiao, H. Yang, M. Lu, Z. You, N. Lu, X. Gao, D. Leggat, H. Okawa, Y. Zhang, Z. Lin, C. Lu, M. Xiao, C. Avila, D. A. Barbosa Trujillo, A. Cabrera, C. Florez, J. Fraga, J. Mejia Guisao, F. Ramirez, M. Rodriguez, J. D. Ruiz Alvarez, D. Giljanovic, N. Godinovic, D. Lelas, I. Puljak, Z. Antunovic, M. Kovac, T. Sculac, V. Brigljevic, B. K. Chitroda, D. Ferencek, S. Mishra, M. Roguljic, A. Starodumov, T. Susa, A. Attikis, K. Christoforou, M. Kolosova, S. Konstantinou, J. Mousa, C. Nicolaou, F. Ptochos, P. A. Razis, H. Rykaczewski, H. Saka, A. Stepennov, M. Finger, M. Finger, A. Kveton, E. Ayala, E. Carrera Jarrin, H. Abdalla, Y. Assran, A. Lotfy, M. A. Mahmoud, S. Bhowmik, R. K. Dewanjee, K. Ehataht, M. Kadastik, T. Lange, S. Nandan, C. Nielsen, J. Pata, M. Raidal, L. Tani, C. Veelken, P. Eerola, H. Kirschenmann, K. Osterberg, M. Voutilainen, S. Bharthuar, E. Brücken, F. Garcia, J. Havukainen, M. S. Kim, R. Kinnunen, T. Lampén, K. Lassila-Perini, S. Lehti, T. Lindén, M. Lotti, L. Martikainen, M. Myllymäki, J. Ott, M. M. Rantanen, H. Siikonen, E. Tuominen, J. Tuominiemi, P. Luukka, H. Petrow, T. Tuuva, C. Amendola, M. Besancon, F. Couderc, M. Dejardin, D. Denegri, J. L. Faure, F. Ferri, S. Ganjour, P. Gras, G. Hamel de Monchenault, V. Lohezic, J. Malcles, J. Rander, A. Rosowsky, M. Ö. Sahin, A. Savoy-Navarro, P. Simkina, M. Titov, C. Baldenegro Barrera, F. Beaudette, A. Buchot Perraguin, P. Busson, A. Cappati, C. Charlot, F. Damas, O. Davignon, B. Diab, G. Falmagne, B. A. Fontana Santos Alves, S. Ghosh, R. Granier de Cassagnac, A. Hakimi, B. Harikrishnan, G. Liu, J. Motta, M. Nguyen, C. Ochando, L. Portales, R. Salerno, U. Sarkar, J. B. Sauvan, Y. Sirois, A. Tarabini, E. Vernazza, A. Zabi, A. Zghiche, J.-L. Agram, J. Andrea, D. Apparu, D. Bloch, G. Bourgatte, J.-M. Brom, E. C. Chabert, C. Collard, D. Darej, U. Goerlach, C. Grimault, A.-C. Le Bihan, P. Van Hove, S. Beauceron, B. Blancon, G. Boudoul, A. Carle, N. Chanon, J. Choi, D. Contardo, P. Depasse, C. Dozen, H. El Mamouni, J. Fay, S. Gascon, M. Gouzevitch, G. Grenier, B. Ille, I. B. Laktineh, M. Lethuillier, L. Mirabito, S. Perries, L. Torterotot, M. Vander Donckt, P. Verdier, S. Viret, D. Chokheli, I. Lomidze, Z. Tsamalaidze, V. Botta, L. Feld, K. Klein, M. Lipinski, D. Meuser, A. Pauls, N. Röwert, M. Teroerde, S. Diekmann, A. Dodonova, N. Eich, D. Eliseev, M. Erdmann, P. Fackeldey, D. Fasanella, B. Fischer, T. Hebbeker, K. Hoepfner, F. Ivone, M. Y. Lee, L. Mastrolorenzo, M. Merschmeyer, A. Meyer, S. Mondal, S. Mukherjee, D. Noll, A. Novak, F. Nowotny, A. Pozdnyakov, Y. Rath, W. Redjeb, H. Reithler, A. Schmidt, S. C. Schuler, A. Sharma, A. Stein, F. Torres Da Silva De Araujo, L. Vigilante, S. Wiedenbeck, S. Zaleski, C. Dziwok, G. Flügge, W. Haj Ahmad, O. Hlushchenko, T. Kress, A. Nowack, O. Pooth, A. Stahl, T. Ziemons, A. Zotz, H. Aarup Petersen, M. Aldaya Martin, P. Asmuss, S. Baxter, M. Bayatmakou, O. Behnke, A. Bermúdez Martínez, S. Bhattacharya, A. A. Bin Anuar, F. Blekman, K. Borras, D. Brunner, A. Campbell, A. Cardini, C. Cheng, F. Colombina, S. Consuegra Rodríguez, G. Correia Silva, M. De Silva, L. Didukh, G. Eckerlin, D. Eckstein, L. I. Estevez Banos, O. Filatov, E. Gallo, A. Geiser, A. Giraldi, G. Greau, A. Grohsjean, V. Guglielmi, M. Guthoff, A. Jafari, N. Z. Jomhari, B. Kaech, M. Kasemann, H. Kaveh, C. Kleinwort, R. Kogler, M. Komm, D. Krücker, W. Lange, D. Leyva Pernia, K. Lipka, W. Lohmann, R. Mankel, I.-A. Melzer-Pellmann, M. Mendizabal Morentin, J. Metwally, A. B. Meyer, G. Milella, M. Mormile, A. Mussgiller, A. Nürnberg, Y. Otarid, D. Pérez Adán, A. Raspereza, B. Ribeiro Lopes, J. Rübenach, A. Saggio, A. Saibel, M. Savitskyi, M. Scham, V. Scheurer, S. Schnake, P. Schütze, C. Schwanenberger, M. Shchedrolosiev, R. E. Sosa Ricardo, D. Stafford, N. Tonon, M. Van De Klundert, F. Vazzoler, A. Ventura Barroso, R. Walsh, D. Walter, Q. Wang, Y. Wen, K. Wichmann, L. Wiens, C. Wissing, S. Wuchterl, Y. Yang, A. Zimermmane Castro Santos, A. Albrecht, S. Albrecht, M. Antonello, S. Bein, L. Benato, M. Bonanomi, P. Connor, K. De Leo, M. Eich, K. El Morabit, F. Feindt, A. Fröhlich, C. Garbers, E. Garutti, M. Hajheidari, J. Haller, A. Hinzmann, H. R. Jabusch, G. Kasieczka, P. Keicher, R. Klanner, W. Korcari, T. Kramer, V. Kutzner, F. Labe, J. Lange, A. Lobanov, C. Matthies, A. Mehta, L. Moureaux, M. Mrowietz, A. Nigamova, Y. Nissan, A. Paasch, K. J. Pena Rodriguez, T. Quadfasel, M. Rieger, O. Rieger, D. Savoiu, J. Schindler, P. Schleper, M. Schröder, J. Schwandt, M. Sommerhalder, H. Stadie, G. Steinbrück, A. Tews, M. Wolf, S. Brommer, M. Burkart, E. Butz, R. Caspart, T. Chwalek, A. Dierlamm, A. Droll, N. Faltermann, M. Giffels, J. O. Gosewisch, A. Gottmann, F. Hartmann, M. Horzela, U. Husemann, M. Klute, R. Koppenhöfer, M. Link, A. Lintuluoto, S. Maier, S. Mitra, Th. Müller, M. Neukum, M. Oh, G. Quast, K. Rabbertz, J. Rauser, M. Schnepf, I. Shvetsov, H. J. Simonis, N. Trevisani, R. Ulrich, J. van der Linden, R. F. Von Cube, M. Wassmer, S. Wieland, R. Wolf, S. Wozniewski, S. Wunsch, X. Zuo, G. Anagnostou, P. Assiouras, G. Daskalakis, A. Kyriakis, A. Stakia, M. Diamantopoulou, D. Karasavvas, P. Kontaxakis, A. Manousakis-Katsikakis, A. Panagiotou, I. Papavergou, N. Saoulidou, K. Theofilatos, E. Tziaferi, K. Vellidis, I. Zisopoulos, G. Bakas, T. Chatzistavrou, K. Kousouris, I. Papakrivopoulos, G. Tsipolitis, A. Zacharopoulou, K. Adamidis, I. Bestintzanos, I. Evangelou, C. Foudas, P. Gianneios, C. Kamtsikis, P. Katsoulis, P. Kokkas, P. G. Kosmoglou Kioseoglou, N. Manthos, I. Papadopoulos, J. Strologas, M. Csanád, K. Farkas, M. M. A. Gadallah, S. Lökös, P. Major, K. Mandal, G. Pásztor, A. J. Rádl, O. Surányi, G. I. Veres, M. Bartók, G. Bencze, C. Hajdu, D. Horvath, F. Sikler, V. Veszpremi, N. Beni, S. Czellar, J. Karancsi, J. Molnar, Z. Szillasi, D. Teyssier, P. Raics, B. Ujvari, T. Csorgo, F. Nemes, T. Novak, J. Babbar, S. Bansal, S. B. Beri, V. Bhatnagar, G. Chaudhary, S. Chauhan, N. Dhingra, R. Gupta, A. Kaur, A. Kaur, H. Kaur, M. Kaur, S. Kumar, P. Kumari, M. Meena, K. Sandeep, T. Sheokand, J. B. Singh, A. Singla, A. K. Virdi, A. Ahmed, A. Bhardwaj, A. Chhetri, B. C. Choudhary, A. Kumar, M. Naimuddin, K. Ranjan, S. Saumya, S. Baradia, S. Barman, S. Bhattacharya, D. Bhowmik, S. Dutta, S. Dutta, B. Gomber, M. Maity, P. Palit, G. Saha, B. Sahu, S. Sarkar, P. K. Behera, S. C. Behera, P. Kalbhor, J. R. Komaragiri, D. Kumar, A. Muhammad, L. Panwar, R. Pradhan, P. R. Pujahari, A. Sharma, A. K. Sikdar, P. C. Tiwari, S. Verma, K. Naskar, T. Aziz, I. Das, S. Dugad, M. Kumar, G. B. Mohanty, P. Suryadevara, S. Banerjee, R. Chudasama, M. Guchait, S. Karmakar, S. Kumar, G. Majumder, K. Mazumdar, S. Mukherjee, A. Thachayath, S. Bahinipati, A. K. Das, C. Kar, P. Mal, T. Mishra, V. K. Muraleedharan Nair Bindhu, A. Nayak, P. Saha, S. K. Swain, D. Vats, A. Alpana, S. Dube, B. Kansal, A. Laha, S. Pandey, A. Rastogi, S. Sharma, H. Bakhshiansohi, E. Khazaie, M. Zeinali, S. Chenarani, S. M. Etesami, M. Khakzad, M. Mohammadi Najafabadi, M. Grunewald, M. Abbrescia, R. Aly, C. Aruta, A. Colaleo, D. Creanza, N. De Filippis, M. De Palma, A. Di Florio, W. Elmetenawee, F. Errico, L. Fiore, G. Iaselli, G. Maggi, M. Maggi, I. Margjeka, V. Mastrapasqua, S. My, S. Nuzzo, A. Pellecchia, A. Pompili, G. Pugliese, R. Radogna, D. Ramos, A. Ranieri, G. Selvaggi, L. Silvestris, F. M. Simone, Ü. Sözbilir, A. Stamerra, R. Venditti, P. Verwilligen, G. Abbiendi, C. Battilana, D. Bonacorsi, L. Borgonovi, R. Campanini, P. Capiluppi, A. Castro, F. R. Cavallo, C. Ciocca, M. Cuffiani, G. M. Dallavalle, T. Diotalevi, F. Fabbri, A. Fanfani, P. Giacomelli, L. Giommi, C. Grandi, L. Guiducci, S. Lo Meo, L. Lunerti, S. Marcellini, G. Masetti, F. L. Navarria, A. Perrotta, F. Primavera, A. M. Rossi, T. Rovelli, G. P. Siroli, S. Costa, A. Di Mattia, R. Potenza, A. Tricomi, C. Tuve, G. Barbagli, G. Bardelli, B. Camaiani, A. Cassese, R. Ceccarelli, V. Ciulli, C. Civinini, R. D’Alessandro, E. Focardi, G. Latino, P. Lenzi, M. Lizzo, M. Meschini, S. Paoletti, R. Seidita, G. Sguazzoni, L. Viliani, L. Benussi, S. Bianco, S. Meola, D. Piccolo, M. Bozzo, P. Chatagnon, F. Ferro, E. Robutti, S. Tosi, A. Benaglia, G. Boldrini, F. Brivio, F. Cetorelli, F. De Guio, M. E. Dinardo, P. Dini, S. Gennai, A. Ghezzi, P. Govoni, L. Guzzi, M. T. Lucchini, M. Malberti, S. Malvezzi, A. Massironi, D. Menasce, L. Moroni, M. Paganoni, D. Pedrini, B. S. Pinolini, S. Ragazzi, N. Redaelli, T. Tabarelli de Fatis, D. Zuolo, S. Buontempo, F. Carnevali, N. Cavallo, A. De Iorio, F. Fabozzi, A. O. M. Iorio, L. Lista, P. Paolucci, B. Rossi, C. Sciacca, P. Azzi, N. Bacchetta, A. Bergnoli, M. Biasotto, D. Bisello, P. Bortignon, A. Bragagnolo, R. Carlin, P. Checchia, T. Dorigo, F. Gasparini, G. Grosso, L. Layer, E. Lusiani, M. Margoni, A. T. Meneguzzo, J. Pazzini, P. Ronchese, R. Rossin, F. Simonetto, G. Strong, M. Tosi, H. Yarar, P. Zotto, A. Zucchetta, G. Zumerle, S. Abu Zeid, C. Aimè, A. Braghieri, S. Calzaferri, D. Fiorina, P. Montagna, V. Re, C. Riccardi, P. Salvini, I. Vai, P. Vitulo, P. Asenov, G. M. Bilei, D. Ciangottini, L. Fanò, M. Magherini, G. Mantovani, V. Mariani, M. Menichelli, F. Moscatelli, A. Piccinelli, M. Presilla, A. Rossi, A. Santocchia, D. Spiga, T. Tedeschi, P. Azzurri, G. Bagliesi, V. Bertacchi, R. Bhattacharya, L. Bianchini, T. Boccali, E. Bossini, D. Bruschini, R. Castaldi, M. A. Ciocci, V. D’Amante, R. Dell’Orso, M. R. Di Domenico, S. Donato, A. Giassi, F. Ligabue, G. Mandorli, D. Matos Figueiredo, A. Messineo, M. Musich, F. Palla, S. Parolia, G. Ramirez-Sanchez, A. Rizzi, G. Rolandi, S. Roy Chowdhury, T. Sarkar, A. Scribano, N. Shafiei, P. Spagnolo, R. Tenchini, G. Tonelli, N. Turini, A. Venturi, P. G. Verdini, P. Barria, M. Campana, F. Cavallari, D. Del Re, E. Di Marco, M. Diemoz, E. Longo, P. Meridiani, G. Organtini, F. Pandolfi, R. Paramatti, C. Quaranta, S. Rahatlou, C. Rovelli, F. Santanastasio, L. Soffi, R. Tramontano, N. Amapane, R. Arcidiacono, S. Argiro, M. Arneodo, N. Bartosik, R. Bellan, A. Bellora, C. Biino, N. Cartiglia, M. Costa, R. Covarelli, N. Demaria, M. Grippo, B. Kiani, F. Legger, C. Mariotti, S. Maselli, A. Mecca, E. Migliore, E. Monteil, M. Monteno, R. Mulargia, M. M. Obertino, G. Ortona, L. Pacher, N. Pastrone, M. Pelliccioni, M. Ruspa, K. Shchelina, F. Siviero, V. Sola, A. Solano, D. Soldi, A. Staiano, M. Tornago, D. Trocino, G. Umoret, A. Vagnerini, S. Belforte, V. Candelise, M. Casarsa, F. Cossutti, A. Da Rold, G. Della Ricca, G. Sorrentino, S. Dogra, C. Huh, B. Kim, D. H. Kim, G. N. Kim, J. Kim, J. Lee, S. W. Lee, C. S. Moon, Y. D. Oh, S. I. Pak, M. S. Ryu, S. Sekmen, Y. C. Yang, H. Kim, D. H. Moon, E. Asilar, T. J. Kim, J. Park, S. Choi, S. Han, B. Hong, K. Lee, K. S. Lee, J. Lim, J. Park, S. K. Park, J. Yoo, J. Goh, H. S. Kim, Y. Kim, S. Lee, J. Almond, J. H. Bhyun, J. Choi, S. Jeon, J. Kim, J. S. Kim, S. Ko, H. Kwon, H. Lee, S. Lee, B. H. Oh, S. B. Oh, H. Seo, U. K. Yang, I. Yoon, W. Jang, D. Y. Kang, Y. Kang, D. Kim, S. Kim, B. Ko, J. S. H. Lee, Y. Lee, J. A. Merlin, I. C. Park, Y. Roh, D. Song, I. J. Watson, S. Yang, S. Ha, H. D. Yoo, M. Choi, M. R. Kim, H. Lee, Y. Lee, Y. Lee, I. Yu, T. Beyrouthy, Y. Maghrbi, K. Dreimanis, G. Pikurs, A. Potrebko, M. Seidel, V. Veckalns, M. Ambrozas, A. Carvalho Antunes De Oliveira, A. Juodagalvis, A. Rinkevicius, G. Tamulaitis, N. Bin Norjoharuddeen, S. Y. Hoh, I. Yusuff, Z. Zolkapli, J. F. Benitez, A. Castaneda Hernandez, H. A. Encinas Acosta, L. G. Gallegos Maríñez, M. León Coello, J. A. Murillo Quijada, A. Sehrawat, L. Valencia Palomo, G. Ayala, H. Castilla-Valdez, I. Heredia-De La Cruz, R. Lopez-Fernandez, C. A. Mondragon Herrera, D. A. Perez Navarro, A. Sánchez Hernández, C. Oropeza Barrera, F. Vazquez Valencia, I. Pedraza, H. A. Salazar Ibarguen, C. Uribe Estrada, I. Bubanja, J. Mijuskovic, N. Raicevic, A. Ahmad, M. I. Asghar, A. Awais, M. I. M. Awan, M. Gul, H. R. Hoorani, W. A. Khan, M. Shoaib, M. Waqas, V. Avati, L. Grzanka, M. Malawski, H. Bialkowska, M. Bluj, B. Boimska, M. Górski, M. Kazana, M. Szleper, P. Zalewski, K. Bunkowski, K. Doroba, A. Kalinowski, M. Konecki, J. Krolikowski, M. Araujo, P. Bargassa, D. Bastos, A. Boletti, P. Faccioli, M. Gallinaro, J. Hollar, N. Leonardo, T. Niknejad, M. Pisano, J. Seixas, J. Varela, P. Adzic, M. Dordevic, P. Milenovic, J. Milosevic, M. Aguilar-Benitez, J. Alcaraz Maestre, A. Álvarez Fernández, M. Barrio Luna, Cristina F. Bedoya, C. A. Carrillo Montoya, M. Cepeda, M. Cerrada, N. Colino, B. De La Cruz, A. Delgado Peris, D. Fernández Del Val, J. P. Fernández Ramos, J. Flix, M. C. Fouz, O. Gonzalez Lopez, S. Goy Lopez, J. M. Hernandez, M. I. Josa, J. León Holgado, D. Moran, C. Perez Dengra, A. Pérez-Calero Yzquierdo, J. Puerta Pelayo, I. Redondo, D. D. Redondo Ferrero, L. Romero, S. Sánchez Navas, J. Sastre, L. Urda Gómez, J. Vazquez Escobar, C. Willmott, J. F. de Trocóniz, B. Alvarez Gonzalez, J. Cuevas, J. Fernandez Menendez, S. Folgueras, I. Gonzalez Caballero, J. R. González Fernández, E. Palencia Cortezon, C. Ramón Álvarez, V. Rodríguez Bouza, A. Soto Rodríguez, A. Trapote, C. Vico Villalba, J. A. Brochero Cifuentes, I. J. Cabrillo, A. Calderon, J. Duarte Campderros, M. Fernandez, C. Fernandez Madrazo, A. García Alonso, G. Gomez, C. Lasaosa García, C. Martinez Rivero, P. Martinez Ruiz del Arbol, F. Matorras, P. Matorras Cuevas, J. Piedra Gomez, C. Prieels, A. Ruiz-Jimeno, L. Scodellaro, I. Vila, J. M. Vizan Garcia, M. K. Jayananda, B. Kailasapathy, D. U. J. Sonnadara, D. D. C. Wickramarathna, W. G. D. Dharmaratna, K. Liyanage, N. Perera, N. Wickramage, D. Abbaneo, J. Alimena, E. Auffray, G. Auzinger, J. Baechler, P. Baillon, D. Barney, J. Bendavid, M. Bianco, B. Bilin, A. Bocci, E. Brondolin, C. Caillol, T. Camporesi, G. Cerminara, N. Chernyavskaya, S. S. Chhibra, S. Choudhury, M. Cipriani, L. Cristella, D. d’Enterria, A. Dabrowski, A. David, A. De Roeck, M. M. Defranchis, M. Deile, M. Dobson, M. Dünser, N. Dupont, F. Fallavollita, A. Florent, L. Forthomme, G. Franzoni, W. Funk, S. Ghosh, S. Giani, D. Gigi, K. Gill, F. Glege, L. Gouskos, E. Govorkova, M. Haranko, J. Hegeman, V. Innocente, T. James, P. Janot, J. Kaspar, J. Kieseler, N. Kratochwil, S. Laurila, P. Lecoq, E. Leutgeb, C. Lourenço, B. Maier, L. Malgeri, M. Mannelli, A. C. Marini, F. Meijers, S. Mersi, E. Meschi, F. Moortgat, M. Mulders, S. Orfanelli, L. Orsini, F. Pantaleo, E. Perez, M. Peruzzi, A. Petrilli, G. Petrucciani, A. Pfeiffer, M. Pierini, D. Piparo, M. Pitt, H. Qu, T. Quast, D. Rabady, A. Racz, G. Reales Gutiérrez, M. Rovere, H. Sakulin, J. Salfeld-Nebgen, S. Scarfi, M. Selvaggi, A. Sharma, P. Silva, P. Sphicas, A. G. Stahl Leiton, S. Summers, K. Tatar, V. R. Tavolaro, D. Treille, P. Tropea, A. Tsirou, J. Wanczyk, K. A. Wozniak, W. D. Zeuner, L. Caminada, A. Ebrahimi, W. Erdmann, R. Horisberger, Q. Ingram, H. C. Kaestli, D. Kotlinski, C. Lange, M. Missiroli, L. Noehte, T. Rohe, T. K. Aarrestad, K. Androsov, M. Backhaus, P. Berger, A. Calandri, K. Datta, A. De Cosa, G. Dissertori, M. Dittmar, M. Donegà, F. Eble, M. Galli, K. Gedia, F. Glessgen, T. A. Gómez Espinosa, C. Grab, D. Hits, W. Lustermann, A.-M. Lyon, R. A. Manzoni, L. Marchese, C. Martin Perez, A. Mascellani, F. Nessi-Tedaldi, J. Niedziela, F. Pauss, V. Perovic, S. Pigazzini, M. G. Ratti, M. Reichmann, C. Reissel, T. Reitenspiess, B. Ristic, F. Riti, D. Ruini, D. A. Sanz Becerra, J. Steggemann, D. Valsecchi, R. Wallny, C. Amsler, P. Bärtschi, C. Botta, D. Brzhechko, M. F. Canelli, K. Cormier, A. De Wit, R. Del Burgo, J. K. Heikkilä, M. Huwiler, W. Jin, A. Jofrehei, B. Kilminster, S. Leontsinis, S. P. Liechti, A. Macchiolo, P. Meiring, V. M. Mikuni, U. Molinatti, I. Neutelings, A. Reimers, P. Robmann, S. Sanchez Cruz, K. Schweiger, M. Senger, Y. Takahashi, C. Adloff, C. M. Kuo, W. Lin, P. K. Rout, S. S. Yu, L. Ceard, Y. Chao, K. F. Chen, P. s. Chen, H. Cheng, W.-S. Hou, R. Khurana, G. Kole, Y. Y. Li, R.-S. Lu, E. Paganis, A. Psallidas, A. Steen, H. y. Wu, E. Yazgan, P. R. Yu, C. Asawatangtrakuldee, N. Srimanobhas, V. Wachirapusitanand, D. Agyel, F. Boran, Z. S. Demiroglu, F. Dolek, I. Dumanoglu, E. Eskut, Y. Guler, E. Gurpinar Guler, C. Isik, O. Kara, A. Kayis Topaksu, U. Kiminsu, G. Onengut, K. Ozdemir, A. Polatoz, A. E. Simsek, B. Tali, U. G. Tok, S. Turkcapar, E. Uslan, I. S. Zorbakir, G. Karapinar, K. Ocalan, M. Yalvac, B. Akgun, I. O. Atakisi, E. Gülmez, M. Kaya, O. Kaya, S. Tekten, A. Cakir, K. Cankocak, Y. Komurcu, S. Sen, O. Aydilek, S. Cerci, B. Hacisahinoglu, I. Hos, B. Isildak, B. Kaynak, S. Ozkorucuklu, C. Simsek, D. Sunar Cerci, B. Grynyov, L. Levchuk, D. Anthony, E. Bhal, J. J. Brooke, A. Bundock, E. Clement, D. Cussans, H. Flacher, M. Glowacki, J. Goldstein, H. F. Heath, L. Kreczko, B. Krikler, S. Paramesvaran, S. Seif El Nasr-Storey, V. J. Smith, N. Stylianou, K. Walkingshaw Pass, R. White, A. H. Ball, K. W. Bell, A. Belyaev, C. Brew, R. M. Brown, D. J. A. Cockerill, C. Cooke, K. V. Ellis, K. Harder, S. Harper, M.-L. Holmberg, Sh. Jain, J. Linacre, K. Manolopoulos, D. M. Newbold, E. Olaiya, D. Petyt, T. Reis, G. Salvi, T. Schuh, C. H. Shepherd-Themistocleous, I. R. Tomalin, T. Williams, R. Bainbridge, P. Bloch, S. Bonomally, J. Borg, C. E. Brown, O. Buchmuller, V. Cacchio, V. Cepaitis, G. S. Chahal, D. Colling, J. S. Dancu, P. Dauncey, G. Davies, J. Davies, M. Della Negra, S. Fayer, G. Fedi, G. Hall, M. H. Hassanshahi, A. Howard, G. Iles, J. Langford, L. Lyons, A.-M. Magnan, S. Malik, A. Martelli, M. Mieskolainen, D. G. Monk, J. Nash, M. Pesaresi, B. C. Radburn-Smith, D. M. Raymond, A. Richards, A. Rose, E. Scott, C. Seez, R. Shukla, A. Tapper, K. Uchida, G. P. Uttley, L. H. Vage, T. Virdee, M. Vojinovic, N. Wardle, S. N. Webb, D. Winterbottom, K. Coldham, J. E. Cole, A. Khan, P. Kyberd, I. D. Reid, S. Abdullin, A. Brinkerhoff, B. Caraway, J. Dittmann, K. Hatakeyama, A. R. Kanuganti, B. McMaster, M. Saunders, S. Sawant, C. Sutantawibul, J. Wilson, R. Bartek, A. Dominguez, R. Uniyal, A. M. Vargas Hernandez, S. I. Cooper, D. Di Croce, S. V. Gleyzer, C. Henderson, C. U. Perez, P. Rumerio, C. West, A. Akpinar, A. Albert, D. Arcaro, C. Cosby, Z. Demiragli, C. Erice, E. Fontanesi, D. Gastler, S. May, J. Rohlf, K. Salyer, D. Sperka, D. Spitzbart, I. Suarez, A. Tsatsos, S. Yuan, G. Benelli, B. Burkle, X. Coubez, D. Cutts, M. Hadley, U. Heintz, J. M. Hogan, T. Kwon, G. Landsberg, K. T. Lau, D. Li, J. Luo, M. Narain, N. Pervan, S. Sagir, F. Simpson, E. Usai, W. Y. Wong, X. Yan, D. Yu, W. Zhang, J. Bonilla, C. Brainerd, R. Breedon, M. Calderon De La Barca Sanchez, M. Chertok, J. Conway, P. T. Cox, R. Erbacher, G. Haza, F. Jensen, O. Kukral, G. Mocellin, M. Mulhearn, D. Pellett, B. Regnery, Y. Yao, F. Zhang, M. Bachtis, R. Cousins, A. Datta, D. Hamilton, J. Hauser, M. Ignatenko, M. A. Iqbal, T. Lam, E. Manca, W. A. Nash, S. Regnard, D. Saltzberg, B. Stone, V. Valuev, R. Clare, J. W. Gary, M. Gordon, G. Hanson, G. Karapostoli, O. R. Long, N. Manganelli, W. Si, S. Wimpenny, J. G. Branson, P. Chang, S. Cittolin, S. Cooperstein, D. Diaz, J. Duarte, R. Gerosa, L. Giannini, J. Guiang, R. Kansal, V. Krutelyov, R. Lee, J. Letts, M. Masciovecchio, F. Mokhtar, M. Pieri, B. V. Sathia Narayanan, V. Sharma, M. Tadel, E. Vourliotis, F. Würthwein, Y. Xiang, A. Yagil, N. Amin, C. Campagnari, M. Citron, G. Collura, A. Dorsett, V. Dutta, J. Incandela, M. Kilpatrick, J. Kim, A. J. Li, P. Masterson, H. Mei, M. Oshiro, M. Quinnan, J. Richman, U. Sarica, R. Schmitz, F. Setti, J. Sheplock, P. Siddireddy, D. Stuart, S. Wang, A. Bornheim, O. Cerri, I. Dutta, A. Latorre, J. M. Lawhorn, J. Mao, H. B. Newman, T. Q. Nguyen, M. Spiropulu, J. R. Vlimant, C. Wang, S. Xie, R. Y. Zhu, J. Alison, S. An, M. B. Andrews, P. Bryant, T. Ferguson, A. Harilal, C. Liu, T. Mudholkar, S. Murthy, M. Paulini, A. Roberts, A. Sanchez, W. Terrill, J. P. Cumalat, W. T. Ford, A. Hassani, G. Karathanasis, E. MacDonald, F. Marini, A. Perloff, C. Savard, N. Schonbeck, K. Stenson, K. A. Ulmer, S. R. Wagner, N. Zipper, J. Alexander, S. Bright-Thonney, X. Chen, D. J. Cranshaw, J. Fan, X. Fan, D. Gadkari, S. Hogan, J. Monroy, J. R. Patterson, D. Quach, J. Reichert, M. Reid, A. Ryd, J. Thom, P. Wittich, R. Zou, M. Albrow, M. Alyari, G. Apollinari, A. Apresyan, L. A. T. Bauerdick, D. Berry, J. Berryhill, P. C. Bhat, K. Burkett, J. N. Butler, A. Canepa, G. B. Cerati, H. W. K. Cheung, F. Chlebana, K. F. Di Petrillo, J. Dickinson, V. D. Elvira, Y. Feng, J. Freeman, A. Gandrakota, Z. Gecse, L. Gray, D. Green, S. Grünendahl, D. Guerrero, O. Gutsche, R. M. Harris, R. Heller, T. C. Herwig, J. Hirschauer, L. Horyn, B. Jayatilaka, S. Jindariani, M. Johnson, U. Joshi, T. Klijnsma, B. Klima, K. H. M. Kwok, S. Lammel, D. Lincoln, R. Lipton, T. Liu, C. Madrid, K. Maeshima, C. Mantilla, D. Mason, P. McBride, P. Merkel, S. Mrenna, S. Nahn, J. Ngadiuba, D. Noonan, V. Papadimitriou, N. Pastika, K. Pedro, C. Pena, F. Ravera, A. Reinsvold Hall, L. Ristori, E. Sexton-Kennedy, N. Smith, A. Soha, L. Spiegel, J. Strait, L. Taylor, S. Tkaczyk, N. V. Tran, L. Uplegger, E. W. Vaandering, I. Zoi, P. Avery, D. Bourilkov, L. Cadamuro, V. Cherepanov, R. D. Field, M. Kim, E. Koenig, J. Konigsberg, A. Korytov, E. Kuznetsova, K. H. Lo, K. Matchev, N. Menendez, G. Mitselmakher, A. Muthirakalayil Madhu, N. Rawal, D. Rosenzweig, S. Rosenzweig, K. Shi, J. Wang, Z. Wu, T. Adams, A. Askew, N. Bower, R. Habibullah, V. Hagopian, T. Kolberg, G. Martinez, H. Prosper, O. Viazlo, M. Wulansatiti, R. Yohay, J. Zhang, M. M. Baarmand, S. Butalla, T. Elkafrawy, M. Hohlmann, R. Kumar Verma, M. Rahmani, F. Yumiceva, M. R. Adams, H. Becerril Gonzalez, R. Cavanaugh, S. Dittmer, O. Evdokimov, C. E. Gerber, D. J. Hofman, D. S. Lemos, A. H. Merrit, C. Mills, G. Oh, T. Roy, S. Rudrabhatla, M. B. Tonjes, N. Varelas, X. Wang, Z. Ye, J. Yoo, M. Alhusseini, K. Dilsiz, L. Emediato, G. Karaman, O. K. Köseyan, J.-P. Merlo, A. Mestvirishvili, J. Nachtman, O. Neogi, H. Ogul, Y. Onel, A. Penzo, C. Snyder, E. Tiras, O. Amram, B. Blumenfeld, L. Corcodilos, J. Davis, A. V. Gritsan, S. Kyriacou, P. Maksimovic, J. Roskes, S. Sekhar, M. Swartz, T. Á. Vámi, A. Abreu, L. F. Alcerro Alcerro, J. Anguiano, P. Baringer, A. Bean, Z. Flowers, T. Isidori, J. King, G. Krintiras, M. Lazarovits, C. Le Mahieu, C. Lindsey, J. Marquez, N. Minafra, M. Murray, M. Nickel, C. Rogan, C. Royon, R. Salvatico, S. Sanders, C. Smith, Q. Wang, G. Wilson, B. Allmond, S. Duric, A. Ivanov, K. Kaadze, A. Kalogeropoulos, D. Kim, Y. Maravin, T. Mitchell, A. Modak, K. Nam, D. Roy, F. Rebassoo, D. Wright, E. Adams, A. Baden, O. Baron, A. Belloni, A. Bethani, S. C. Eno, N. J. Hadley, S. Jabeen, R. G. Kellogg, T. Koeth, Y. Lai, S. Lascio, A. C. Mignerey, S. Nabili, C. Palmer, C. Papageorgakis, L. Wang, K. Wong, D. Abercrombie, W. Busza, I. A. Cali, Y. Chen, M. D’Alfonso, J. Eysermans, C. Freer, G. Gomez-Ceballos, M. Goncharov, P. Harris, M. Hu, D. Kovalskyi, J. Krupa, Y.-J. Lee, K. Long, C. Mironov, C. Paus, D. Rankin, C. Roland, G. Roland, Z. Shi, G. S. F. Stephans, J. Wang, Z. Wang, B. Wyslouch, T. J. Yang, R. M. Chatterjee, B. Crossman, A. Evans, J. Hiltbrand, B. M. Joshi, C. Kapsiak, M. Krohn, Y. Kubota, J. Mans, M. Revering, R. Rusack, R. Saradhy, N. Schroeder, N. Strobbe, M. A. Wadud, L. M. Cremaldi, K. Bloom, M. Bryson, D. R. Claes, C. Fangmeier, L. Finco, F. Golf, C. Joo, R. Kamalieddin, I. Kravchenko, I. Reed, J. E. Siado, G. R. Snow, W. Tabb, A. Wightman, F. Yan, A. G. Zecchinelli, G. Agarwal, H. Bandyopadhyay, L. Hay, I. Iashvili, A. Kharchilava, C. McLean, M. Morris, D. Nguyen, J. Pekkanen, S. Rappoccio, A. Williams, G. Alverson, E. Barberis, Y. Haddad, Y. Han, A. Krishna, J. Li, J. Lidrych, G. Madigan, B. Marzocchi, D. M. Morse, V. Nguyen, T. Orimoto, A. Parker, L. Skinnari, A. Tishelman-Charny, T. Wamorkar, B. Wang, A. Wisecarver, D. Wood, S. Bhattacharya, J. Bueghly, Z. Chen, A. Gilbert, K. A. Hahn, Y. Liu, N. Odell, M. H. Schmitt, M. Velasco, R. Band, R. Bucci, M. Cremonesi, A. Das, R. Goldouzian, M. Hildreth, K. Hurtado Anampa, C. Jessop, K. Lannon, J. Lawrence, N. Loukas, L. Lutton, J. Mariano, N. Marinelli, I. Mcalister, T. McCauley, C. Mcgrady, K. Mohrman, C. Moore, Y. Musienko, R. Ruchti, A. Townsend, M. Wayne, H. Yockey, M. Zarucki, L. Zygala, B. Bylsma, M. Carrigan, L. S. Durkin, B. Francis, C. Hill, M. Joyce, A. Lesauvage, M. Nunez Ornelas, K. Wei, B. L. Winer, B. R. Yates, F. M. Addesa, P. Das, G. Dezoort, P. Elmer, A. Frankenthal, B. Greenberg, N. Haubrich, S. Higginbotham, G. Kopp, S. Kwan, D. Lange, D. Marlow, I. Ojalvo, J. Olsen, D. Stickland, C. Tully, S. Malik, S. Norberg, A. S. Bakshi, V. E. Barnes, R. Chawla, S. Das, L. Gutay, M. Jones, A. W. Jung, D. Kondratyev, A. M. Koshy, M. Liu, G. Negro, N. Neumeister, G. Paspalaki, S. Piperov, A. Purohit, J. F. Schulte, M. Stojanovic, J. Thieman, F. Wang, R. Xiao, W. Xie, J. Dolen, N. Parashar, D. Acosta, A. Baty, T. Carnahan, S. Dildick, K. M. Ecklund, P. J. Fernández Manteca, S. Freed, P. Gardner, F. J. M. Geurts, A. Kumar, W. Li, B. P. Padley, R. Redjimi, J. Rotter, S. Yang, E. Yigitbasi, L. Zhang, Y. Zhang, A. Bodek, P. de Barbaro, R. Demina, J. L. Dulemba, C. Fallon, T. Ferbel, M. Galanti, A. Garcia-Bellido, O. Hindrichs, A. Khukhunaishvili, P. Parygin, E. Popova, E. Ranken, R. Taus, G. P. Van Onsem, K. Goulianos, B. Chiarito, J. P. Chou, Y. Gershtein, E. Halkiadakis, A. Hart, M. Heindl, D. Jaroslawski, O. Karacheban, I. Laflotte, A. Lath, R. Montalvo, K. Nash, M. Osherson, H. Routray, S. Salur, S. Schnetzer, S. Somalwar, R. Stone, S. A. Thayil, S. Thomas, H. Wang, H. Acharya, A. G. Delannoy, S. Fiorendi, T. Holmes, E. Nibigira, S. Spanier, O. Bouhali, M. Dalchenko, A. Delgado, R. Eusebi, J. Gilmore, T. Huang, T. Kamon, H. Kim, S. Luo, S. Malhotra, R. Mueller, D. Overton, D. Rathjens, A. Safonov, N. Akchurin, J. Damgov, V. Hegde, K. Lamichhane, S. W. Lee, T. Mengke, S. Muthumuni, T. Peltola, I. Volobouev, A. Whitbeck, E. Appelt, S. Greene, A. Gurrola, W. Johns, A. Melo, F. Romeo, P. Sheldon, S. Tuo, J. Velkovska, J. Viinikainen, B. Cardwell, B. Cox, G. Cummings, J. Hakala, R. Hirosky, A. Ledovskoy, A. Li, C. Neu, C. E. Perez Lara, B. Tannenwald, P. E. Karchin, N. Poudyal, S. Banerjee, K. Black, T. Bose, S. Dasu, I. De Bruyn, P. Everaerts, C. Galloni, H. He, M. Herndon, A. Herve, C. K. Koraka, A. Lanaro, A. Loeliger, R. Loveless, J. Madhusudanan Sreekala, A. Mallampalli, A. Mohammadi, S. Mondal, G. Parida, D. Pinna, A. Savin, V. Shang, V. Sharma, W. H. Smith, D. Teague, H. F. Tsoi, W. Vetens, S. Afanasiev, V. Andreev, Yu. Andreev, T. Aushev, M. Azarkin, A. Babaev, A. Belyaev, V. Blinov, E. Boos, V. Borshch, D. Budkouski, V. Bunichev, V. Chekhovsky, R. Chistov, M. Danilov, A. Dermenev, T. Dimova, I. Dremin, M. Dubinin, L. Dudko, V. Epshteyn, G. Gavrilov, V. Gavrilov, S. Gninenko, V. Golovtcov, N. Golubev, I. Golutvin, I. Gorbunov, A. Gribushin, Y. Ivanov, V. Kachanov, L. Kardapoltsev, V. Karjavine, A. Karneyeu, V. Kim, M. Kirakosyan, D. Kirpichnikov, M. Kirsanov, V. Klyukhin, D. Konstantinov, V. Korenkov, A. Kozyrev, N. Krasnikov, A. Lanev, P. Levchenko, A. Litomin, N. Lychkovskaya, V. Makarenko, A. Malakhov, V. Matveev, V. Murzin, A. Nikitenko, S. Obraztsov, A. Oskin, I. Ovtin, V. Palichik, V. Perelygin, M. Perfilov, S. Petrushanko, S. Polikarpov, V. Popov, O. Radchenko, M. Savina, V. Savrin, V. Shalaev, S. Shmatov, S. Shulha, Y. Skovpen, S. Slabospitskii, V. Smirnov, D. Sosnov, V. Sulimov, E. Tcherniaev, A. Terkulov, O. Teryaev, I. Tlisova, M. Toms, A. Toropin, L. Uvarov, A. Uzunian, E. Vlasov, P. Volkov, A. Vorobyev, N. Voytishin, B. S. Yuldashev, A. Zarubin, I. Zhizhin, A. Zhokin, CMS Collaboration

**Affiliations:** 1 Yerevan Physics Institute, Yerevan, Armenia; 2 Institut für Hochenergiephysik, Vienna, Austria; 3 Universiteit Antwerpen, Antwerpen, Belgium; 4 Vrije Universiteit Brussel, Brussels, Belgium; 5 Université Libre de Bruxelles, Brussels, Belgium; 6 Ghent University, Ghent, Belgium; 7 Université Catholique de Louvain, Louvain-la-Neuve, Belgium; 8 Centro Brasileiro de Pesquisas Fisicas, Rio de Janeiro, Brazil; 9 Universidade do Estado do Rio de Janeiro, Rio de Janeiro, Brazil; 10 Universidade Estadual Paulista, Universidade Federal do ABC, São Paulo, Brazil; 11 Institute for Nuclear Research and Nuclear Energy, Bulgarian Academy of Sciences, Sofia, Bulgaria; 12 University of Sofia, Sofia, Bulgaria; 13 Instituto De Alta Investigación, Universidad de Tarapacá, Casilla 7 D, Arica, Chile; 14 Beihang University, Beijing, China; 15 Department of Physics, Tsinghua University, Beijing, China; 16 Institute of High Energy Physics, Beijing, China; 17 State Key Laboratory of Nuclear Physics and Technology, Peking University, Beijing, China; 18 Sun Yat-Sen University, Guangzhou, China; 19 University of Science and Technology of China, Hefei, China; 20 Institute of Modern Physics and Key Laboratory of Nuclear Physics and Ion-beam Application (MOE), Fudan University, Shanghai, China; 21 Zhejiang University, Hangzhou, Zhejiang China; 22 Universidad de Los Andes, Bogotá, Colombia; 23 Universidad de Antioquia, Medellín, Colombia; 24 Faculty of Electrical Engineering, Mechanical Engineering and Naval Architecture, University of Split, Split, Croatia; 25 Faculty of Science, University of Split, Split, Croatia; 26 Institute Rudjer Boskovic, Zagreb, Croatia; 27 University of Cyprus, Nicosia, Cyprus; 28 Charles University, Prague, Czech Republic; 29 Escuela Politecnica Nacional, Quito, Ecuador; 30 Universidad San Francisco de Quito, Quito, Ecuador; 31 Academy of Scientific Research and Technology of the Arab Republic of Egypt, Egyptian Network of High Energy Physics, Cairo, Egypt; 32 Center for High Energy Physics (CHEP-FU), Fayoum University, El-Fayoum, Egypt; 33 National Institute of Chemical Physics and Biophysics, Tallinn, Estonia; 34 Department of Physics, University of Helsinki, Helsinki, Finland; 35 Helsinki Institute of Physics, Helsinki, Finland; 36 Lappeenranta-Lahti University of Technology, Lappeenranta, Finland; 37 IRFU, CEA, Université Paris-Saclay, Gif-sur-Yvette, France; 38 Laboratoire Leprince-Ringuet, CNRS/IN2P3, Ecole Polytechnique, Institut Polytechnique de Paris, Palaiseau, France; 39 Université de Strasbourg, CNRS, IPHC UMR 7178, Strasbourg, France; 40 Institut de Physique des 2 Infinis de Lyon (IP2I), Villeurbanne, France; 41 Georgian Technical University, Tbilisi, Georgia; 42 I. Physikalisches Institut, RWTH Aachen University, Aachen, Germany; 43 III. Physikalisches Institut A, RWTH Aachen University, Aachen, Germany; 44 III. Physikalisches Institut B, RWTH Aachen University, Aachen, Germany; 45 Deutsches Elektronen-Synchrotron, Hamburg, Germany; 46 University of Hamburg, Hamburg, Germany; 47 Karlsruher Institut für Technologie, Karlsruhe, Germany; 48 Institute of Nuclear and Particle Physics (INPP), NCSR Demokritos, Aghia Paraskevi, Greece; 49 National and Kapodistrian University of Athens, Athens, Greece; 50 National Technical University of Athens, Athens, Greece; 51 University of Ioánnina, Ioannina, Greece; 52 MTA-ELTE Lendület CMS Particle and Nuclear Physics Group, Eötvös Loránd University, Budapest, Hungary; 53 Wigner Research Centre for Physics, Budapest, Hungary; 54 Institute of Nuclear Research ATOMKI, Debrecen, Hungary; 55 Institute of Physics, University of Debrecen, Debrecen, Hungary; 56 Karoly Robert Campus, MATE Institute of Technology, Gyongyos, Hungary; 57 Panjab University, Chandigarh, India; 58 University of Delhi, Delhi, India; 59 Saha Institute of Nuclear Physics, HBNI, Kolkata, India; 60 Indian Institute of Technology Madras, Chennai, India; 61 Bhabha Atomic Research Centre, Mumbai, India; 62 Tata Institute of Fundamental Research-A, Mumbai, India; 63 Tata Institute of Fundamental Research-B, Mumbai, India; 64 National Institute of Science Education and Research, An OCC of Homi Bhabha National Institute, Bhubaneswar, Odisha India; 65 Indian Institute of Science Education and Research (IISER), Pune, India; 66 Isfahan University of Technology, Isfahan, Iran; 67 Institute for Research in Fundamental Sciences (IPM), Tehran, Iran; 68 University College Dublin, Dublin, Ireland; 69 INFN Sezione di Bari, Università di Bari, Politecnico di Bari, Bari, Italy; 70 INFN Sezione di Bologna, Università di Bologna, Bologna, Italy; 71 INFN Sezione di Catania, Università di Catania, Catania, Italy; 72 INFN Sezione di Firenze, Università di Firenze, Florence, Italy; 73 INFN Laboratori Nazionali di Frascati, Frascati, Italy; 74 INFN Sezione di Genova, Università di Genova, Genoa, Italy; 75 INFN Sezione di Milano-Bicocca, Università di Milano-Bicocca, Milan, Italy; 76 INFN Sezione di Napoli, Università di Napoli ‘Federico II’, Napoles, Italy; Università della Basilicata, Potenza, Italy; Università G. Marconi, Rome, Italy; 77 INFN Sezione di Padova, Università di Padova, Padua, Italy; Università di Trento, Trento, Italy; 78 INFN Sezione di Pavia, Università di Pavia, Pavia, Italy; 79 INFN Sezione di Perugia, Università di Perugia, Perugia, Italy; 80 INFN Sezione di Pisa, Università di Pisa, Scuola Normale Superiore di Pisa, Pisa, Italy; Università di Siena, Siena, Italy; 81 INFN Sezione di Roma, Sapienza Università di Roma, Rome, Italy; 82 INFN Sezione di Torino, Università di Torino, Turin, Italy; Università del Piemonte Orientale, Novara, Italy; 83 INFN Sezione di Trieste, Università di Trieste, Trieste, Italy; 84 Kyungpook National University, Daegu, Korea; 85 Institute for Universe and Elementary Particles, Chonnam National University, Kwangju, Korea; 86 Hanyang University, Seoul, Korea; 87 Korea University, Seoul, Korea; 88 Department of Physics, Kyung Hee University, Seoul, Korea; 89 Sejong University, Seoul, Korea; 90 Seoul National University, Seoul, Korea; 91 University of Seoul, Seoul, Korea; 92 Department of Physics, Yonsei University, Seoul, Korea; 93 Sungkyunkwan University, Suwon, Korea; 94 College of Engineering and Technology, American University of the Middle East (AUM), Dasman, Kuwait; 95 Riga Technical University, Riga, Latvia; 96 Vilnius University, Vilnius, Lithuania; 97 National Centre for Particle Physics, Universiti Malaya, Kuala Lumpur, Malaysia; 98 Universidad de Sonora (UNISON), Hermosillo, Mexico; 99 Centro de Investigacion y de Estudios Avanzados del IPN, Mexico City, Mexico; 100 Universidad Iberoamericana, Mexico City, Mexico; 101 Benemerita Universidad Autonoma de Puebla, Puebla, Mexico; 102 University of Montenegro, Podgorica, Montenegro; 103 National Centre for Physics, Quaid-I-Azam University, Islamabad, Pakistan; 104 Faculty of Computer Science, Electronics and Telecommunications, AGH University of Krakow, Krakow, Poland; 105 National Centre for Nuclear Research, Swierk, Poland; 106 Institute of Experimental Physics, Faculty of Physics, University of Warsaw, Warsaw, Poland; 107 Laboratório de Instrumentação e Física Experimental de Partículas, Lisbon, Portugal; 108 VINCA Institute of Nuclear Sciences, University of Belgrade, Belgrade, Serbia; 109 Centro de Investigaciones Energéticas Medioambientales y Tecnológicas (CIEMAT), Madrid, Spain; 110 Universidad Autónoma de Madrid, Madrid, Spain; 111 Instituto Universitario de Ciencias y Tecnologías Espaciales de Asturias (ICTEA), Universidad de Oviedo, Oviedo, Spain; 112 Instituto de Física de Cantabria (IFCA), CSIC-Universidad de Cantabria, Santander, Spain; 113 University of Colombo, Colombo, Sri Lanka; 114 Department of Physics, University of Ruhuna, Matara, Sri Lanka; 115 CERN, European Organization for Nuclear Research, Geneva, Switzerland; 116 Paul Scherrer Institut, Villigen, Switzerland; 117 ETH Zurich-Institute for Particle Physics and Astrophysics (IPA), Zurich, Switzerland; 118 Universität Zürich, Zurich, Switzerland; 119 National Central University, Chung-Li, Taiwan; 120 National Taiwan University (NTU), Taipei, Taiwan; 121 Department of Physics, Faculty of Science, Chulalongkorn University, Bangkok, Thailand; 122 Physics Department, Science and Art Faculty, Çukurova University, Adana, Turkey; 123 Physics Department, Middle East Technical University, Ankara, Turkey; 124 Bogazici University, Istanbul, Turkey; 125 Istanbul Technical University, Istanbul, Turkey; 126 Istanbul University, Istanbul, Turkey; 127 Institute for Scintillation Materials of National Academy of Science of Ukraine, Kharkiv, Ukraine; 128 National Science Centre, Kharkiv Institute of Physics and Technology, Kharkiv, Ukraine; 129 University of Bristol, Bristol, UK; 130 Rutherford Appleton Laboratory, Didcot, UK; 131 Imperial College, London, UK; 132 Brunel University, Uxbridge, UK; 133 Baylor University, Waco, TX USA; 134 Catholic University of America, Washington, DC USA; 135 The University of Alabama, Tuscaloosa, AL USA; 136 Boston University, Boston, MA USA; 137 Brown University, Providence, RI USA; 138 University of California, Davis, Davis, CA USA; 139 University of California, Los Angeles, CA USA; 140 University of California, Riverside, Riverside, CA USA; 141 University of California, San Diego, La Jolla, CA USA; 142 Department of Physics, University of California, Santa Barbara, Santa Barbara, CA USA; 143 California Institute of Technology, Pasadena, CA USA; 144 Carnegie Mellon University, Pittsburgh, Pennsylvania USA; 145 University of Colorado Boulder, Boulder, CO USA; 146 Cornell University, Ithaca, NY USA; 147 Fermi National Accelerator Laboratory, Batavia, IL USA; 148 University of Florida, Gainesville, FL USA; 149 Florida State University, Tallahassee, FL USA; 150 Florida Institute of Technology, Melbourne, FL USA; 151 University of Illinois Chicago, Chicago, USA; 152 The University of Iowa, Iowa City, IA USA; 153 Johns Hopkins University, Baltimore, MD USA; 154 The University of Kansas, Lawrence, KS USA; 155 Kansas State University, Manhattan, KS USA; 156 Lawrence Livermore National Laboratory, Livermore, CA USA; 157 University of Maryland, College Park, MD USA; 158 Massachusetts Institute of Technology, Cambridge, MA USA; 159 University of Minnesota, Minneapolis, MN USA; 160 University of Mississippi, Oxford, MS USA; 161 University of Nebraska-Lincoln, Lincoln, NE USA; 162 State University of New York at Buffalo, Buffalo, NY USA; 163 Northeastern University, Boston, MA USA; 164 Northwestern University, Evanston, IL USA; 165 University of Notre Dame, Notre Dame, IN USA; 166 The Ohio State University, Columbus, OH USA; 167 Princeton University, Princeton, NJ USA; 168 University of Puerto Rico, Mayagüez, PR USA; 169 Purdue University, West Lafayette, IN USA; 170 Purdue University Northwest, Hammond, IN USA; 171 Rice University, Houston, TX USA; 172 University of Rochester, Rochester, NY USA; 173 The Rockefeller University, New York, NY USA; 174 Rutgers, The State University of New Jersey, Piscataway, NJ USA; 175 University of Tennessee, Knoxville, TN USA; 176 Texas A &M University, College Station, TX USA; 177 Texas Tech University, Lubbock, TX USA; 178 Vanderbilt University, Nashville, TN USA; 179 University of Virginia, Charlottesville, VA USA; 180 Wayne State University, Detroit, MI USA; 181 University of Wisconsin-Madison, Madison, WI USA; 182 Authors Affiliated with an Institute or an International Laboratory Covered by a Cooperation Agreement with CERN, Geneva, Switzerland; 183 Yerevan State University, Yerevan, Armenia; 184 TU Wien, Vienna, Austria; 185 Institute of Basic and Applied Sciences, Faculty of Engineering, Arab Academy for Science, Technology and Maritime Transport, Alexandria, Egypt; 186 Université Libre de Bruxelles, Brussels, Belgium; 187 Universidade Estadual de Campinas, Campinas, Brazil; 188 Federal University of Rio Grande do Sul, Porto Alegre, Brazil; 189 UFMS, Nova Andradina, Brazil; 190 University of Chinese Academy of Sciences, Beijing, China; 191 Nanjing Normal University, Nanjing, China; 192 The University of Iowa, Iowa City, IA USA; 193 University of Chinese Academy of Sciences, Beijing, China; 194 an Institute or an International Laboratory Covered by a Cooperation Agreement with CERN, Geneva, Switzerland; 195 Cairo University, Cairo, Egypt; 196 Suez University, Suez, Egypt; 197 British University in Egypt, Cairo, Egypt; 198 Purdue University, West Lafayette, IN USA; 199 Université de Haute Alsace, Mulhouse, France; 200 Department of Physics, Tsinghua University, Beijing, China; 201 The University of the State of Amazonas, Manaus, Brazil; 202 Erzincan Binali Yildirim University, Erzincan, Turkey; 203 University of Hamburg, Hamburg, Germany; 204 III. Physikalisches Institut A, RWTH Aachen University, Aachen, Germany; 205 Isfahan University of Technology, Isfahan, Iran; 206 Bergische University Wuppertal (BUW), Wuppertal, Germany; 207 Brandenburg University of Technology, Cottbus, Germany; 208 Forschungszentrum Jülich, Jülich, Germany; 209 CERN, European Organization for Nuclear Research, Geneva, Switzerland; 210 Physics Department, Faculty of Science, Assiut University, Assiut, Egypt; 211 Karoly Robert Campus, MATE Institute of Technology, Gyongyos, Hungary; 212 Wigner Research Centre for Physics, Budapest, Hungary; 213 Institute of Physics, University of Debrecen, Debrecen, Hungary; 214 Institute of Nuclear Research ATOMKI, Debrecen, Hungary; 215 Facultatea de Fizica, Universitatea Babes-Bolyai, Cluj-Napoca, Romania; 216 Faculty of Informatics, University of Debrecen, Debrecen, Hungary; 217 Punjab Agricultural University, Ludhiana, India; 218 UPES-University of Petroleum and Energy Studies, Dehra Dun, India; 219 University of Visva-Bharati, Santiniketan, India; 220 University of Hyderabad, Hyderabad, India; 221 Indian Institute of Science (IISc), Bangalore, India; 222 Indian Institute of Technology (IIT), Mumbai, India; 223 IIT Bhubaneswar, Bhubaneswar, India; 224 Institute of Physics, Bhubaneswar, India; 225 Deutsches Elektronen-Synchrotron, Hamburg, Germany; 226 Department of Physics, Isfahan University of Technology, Isfahan, Iran; 227 Sharif University of Technology, Tehran, Iran; 228 Department of Physics, University of Science and Technology of Mazandaran, Behshahr, Iran; 229 Helwan University, Cairo, Egypt; 230 Italian National Agency for New Technologies, Energy and Sustainable Economic Development, Bologna, Italy; 231 Centro Siciliano di Fisica Nucleare e di Struttura Della Materia, Catania, Italy; 232 Università degli Studi Guglielmo Marconi, Rome, Italy; 233 Scuola Superiore Meridionale, Università di Napoli ‘Federico II’, Naples, Italy; 234 Fermi National Accelerator Laboratory, Batavia, IL USA; 235 Laboratori Nazionali di Legnaro dell’INFN, Legnaro, Italy; 236 Università di Napoli ‘Federico II’, Naples, Italy; 237 Ain Shams University, Cairo, Egypt; 238 Consiglio Nazionale delle Ricerche-Istituto Officina dei Materiali, Perugia, Italy; 239 Department of Applied Physics, Faculty of Science and Technology, Universiti Kebangsaan Malaysia, Bangi, Malaysia; 240 Consejo Nacional de Ciencia y Tecnología, Mexico City, Mexico; 241 IRFU, CEA, Université Paris-Saclay, Gif-sur-Yvette, France; 242 Faculty of Physics, University of Belgrade, Belgrade, Serbia; 243 Trincomalee Campus, Eastern University, Sri Lanka, Nilaveli, Sri Lanka; 244 Saegis Campus, Nugegoda, Sri Lanka; 245 INFN Sezione di Pavia, Università di Pavia, Pavia, Italy; 246 National and Kapodistrian University of Athens, Athens, Greece; 247 Ecole Polytechnique Fédérale Lausanne, Lausanne, Switzerland; 248 Universität Zürich, Zurich, Switzerland; 249 Stefan Meyer Institute for Subatomic Physics, Vienna, Austria; 250 Laboratoire d’Annecy-le-Vieux de Physique des Particules, IN2P3-CNRS, Annecy-le-Vieux, France; 251 Research Center of Experimental Health Science, Near East University, Mersin, Turkey; 252 Konya Technical University, Konya, Turkey; 253 Izmir Bakircay University, Izmir, Turkey; 254 Adiyaman University, Adiyaman, Turkey; 255 Istanbul Gedik University, Istanbul, Turkey; 256 Necmettin Erbakan University, Konya, Turkey; 257 Bozok Universitetesi Rektörlügü, Yozgat, Turkey; 258 Marmara University, Istanbul, Turkey; 259 Milli Savunma University, Istanbul, Turkey; 260 Kafkas University, Kars, Turkey; 261 Hacettepe University, Ankara, Turkey; 262 Faculty of Engineering, Istanbul University-Cerrahpasa, Istanbul, Turkey; 263 Ozyegin University, Istanbul, Turkey; 264 Vrije Universiteit Brussel, Brussels, Belgium; 265 School of Physics and Astronomy, University of Southampton, Southampton, UK; 266 University of Bristol, Bristol, UK; 267 IPPP Durham University, Durham, UK; 268 Faculty of Science, Monash University, Clayton, Australia; 269 Università di Torino, Turin, Italy; 270 Bethel University, St. Paul, MN USA; 271 Karamanoğlu Mehmetbey University, Karaman, Turkey; 272 California Institute of Technology, Pasadena, CA USA; 273 United States Naval Academy, Annapolis, MD USA; 274 Bingol University, Bingol, Turkey; 275 Georgian Technical University, Tbilisi, Georgia; 276 Sinop University, Sinop, Turkey; 277 Erciyes University, Kayseri, Turkey; 278 Institute of Modern Physics and Key Laboratory of Nuclear Physics and Ion-beam Application (MOE), Fudan University, Shanghai, China; 279 Texas A &M University at Qatar, Doha, Qatar; 280 Kyungpook National University, Daegu, Korea; 281 Another Institute or International Laboratory Covered by a Cooperation Agreement with CERN, Geneva, Switzerland; 282 Imperial College, London, UK; 283 Institute of Nuclear Physics of the Uzbekistan Academy of Sciences, Tashkent, Uzbekistan; 284 CERN, Geneva, Switzerland

## Abstract

The mass of the top quark is measured in 36.3$\,\text {fb}^{-1}$ of LHC proton–proton collision data collected with the CMS detector at $\sqrt{s}=13\,\text {Te}\hspace{-.08em}\text {V}$. The measurement uses a sample of top quark pair candidate events containing one isolated electron or muon and at least four jets in the final state. For each event, the mass is reconstructed from a kinematic fit of the decay products to a top quark pair hypothesis. A profile likelihood method is applied using up to four observables per event to extract the top quark mass. The top quark mass is measured to be $171.77\pm 0.37\,\text {Ge}\hspace{-.08em}\text {V}$. This approach significantly improves the precision over previous measurements.

## Introduction

The top quark [1, 2] is the most massive fundamental particle and its mass, $m_{\textrm{t}}$, is an important free parameter of the standard model (SM) of particle physics. Because of its large Yukawa coupling, the top quark dominates the higher-order corrections to the Higgs boson mass and a precise determination of $m_{\textrm{t}}$ sets strong constraints on the stability of the electroweak vacuum [3, 4]. In addition, precise measurements of $m_{\textrm{t}}$ can be used to test the internal consistency of the SM [5–7].

At the CERN LHC, top quarks are produced predominantly in quark–antiquark pairs ($\hbox {t}\bar{\hbox {t}}$), which decay almost exclusively into a bottom (b) quark and a W boson. Each $\hbox {t}\bar{\hbox {t}}$ event can be classified by the subsequent decay of the W bosons. For this paper, the lepton + jets channel is analyzed, where one W boson decays hadronically, and the other leptonically. Hence, the minimal final state consists of a muon or electron, at least four jets, and one undetected neutrino. This includes events where a muon or electron from a tau lepton decay passes the selection criteria.

The mass of the top quark has been measured with increasing precision using the reconstructed invariant mass of different combinations of its decay products [8]. The measurements by the Tevatron collaborations led to a combined value of $m_{\textrm{t}} =174.30\pm 0.65\,\text {Ge}\hspace{-.08em}\text {V}$ [9], while the ATLAS and CMS Collaborations measured $m_{\textrm{t}} =172.69\pm 0.48\,\text {Ge}\hspace{-.08em}\text {V}$ [10] and $m_{\textrm{t}} =172.44\pm 0.48\,\text {Ge}\hspace{-.08em}\text {V}$ [11], respectively, from the combination of their most precise results at $\sqrt{s}=7$ and 8 TeV (Run 1). The LHC measurements achieved a relative precision on $m_{\textrm{t}}$ of 0.28%. These analyses extract $m_{\textrm{t}}$ by comparing data directly to Monte Carlo simulations for different values of $m_{\textrm{t}}$. An overview of the discussion of this mass definition and its relationship to a theoretically well-defined parameter is presented in Ref. [12].

In the lepton + jets channel, $m_{\textrm{t}}$ was measured by the CMS Collaboration with proton–proton (pp) collision data at $\sqrt{s} = 13\,\text {Te}\hspace{-.08em}\text {V}$. The result of $m_{\textrm{t}} = 172.25\pm 0.63\,\text {Ge}\hspace{-.08em}\text {V}$ [13] was extracted using the ideogram method [14, 15], which had previously been employed in Run 1 [11]. In contrast to the Run 1 analysis, in the analysis of $\sqrt{s} = 13\,\text {Te}\hspace{-.08em}\text {V}$ data, the renormalization and factorization scales in the matrix-element (ME) calculation and the scales in the initial- and final-state parton showers (PS) were varied separately, in order to evaluate the corresponding systematic uncertainties. In addition, the impacts of extended models of color reconnection (CR) were evaluated. These models were not available for the Run 1 measurements and their inclusion resulted in an increase in the systematic uncertainty [13].

In this paper, we use a new mass extraction method on the same data, corresponding to 36.3$\,\text {fb}^{-1}$, that were used in Ref. [13]. In addition to developments in the mass extraction technique, the reconstruction and calibration of the analyzed data have been improved, and updated simulations are used. For example, the underlying event tune CP5 [16] and the jet flavor tagger DeepJet [17] were not available in the former analysis on the data. The new analysis employs a kinematic fit of the decay products to a $\hbox {t}\bar{\hbox {t}}$ hypothesis. For each event, the best matching assignment of the jets to the decay products is used. A profile likelihood fit is performed using up to four different observables for events that are well reconstructed by the kinematic fit and one observable for the remaining events. The additional observables are used to constrain the main sources of systematic uncertainty. The model for the likelihood incorporates the effects of variations of these sources, represented by nuisance parameters based on simulation, as well as the finite size of the simulated samples. This reduces the influence of systematic uncertainties in the measurement. Tabulated results are provided in the HEPData record for this analysis [18].

## The CMS detector and event reconstruction

The central feature of the CMS apparatus is a superconducting solenoid of 6$\,\text {m}$ internal diameter, which provides a magnetic field of 3.8$\,\text {T}$. Within the solenoid volume are a silicon pixel and strip tracker, a lead tungstate crystal electromagnetic calorimeter (ECAL), and a brass and scintillator hadron calorimeter (HCAL), each composed of a barrel and two endcap sections. Forward calorimeters extend the pseudorapidity ($\eta$) coverage provided by the barrel and endcap detectors. Muons are measured in gas-ionization detectors embedded in the steel flux-return yoke outside the solenoid. A more detailed description of the CMS detector, together with a definition of the coordinate system used and the relevant kinematic variables, can be found in Ref. [19].

The primary vertex is taken to be the vertex corresponding to the hardest scattering in the event, evaluated using tracking information alone, as described in Section 9.4.1 of Ref. [20]. The particle-flow (PF) algorithm [21] aims to reconstruct and identify each individual particle in an event, with an optimized combination of information from the various elements of the CMS detector. The energy of photons is obtained from the ECAL measurement. The energy of electrons is determined from a combination of the electron momentum at the primary interaction vertex as determined by the tracker, the energy of the corresponding ECAL cluster, and the energy sum of all bremsstrahlung photons spatially compatible with originating from the electron track. The energy of muons is obtained from the curvature of the corresponding track. The energy of charged hadrons is determined from a combination of their momentum measured in the tracker and the matching ECAL and HCAL energy deposits, corrected for the response function of the calorimeters to hadronic showers. Finally, the energy of neutral hadrons is obtained from the corresponding corrected ECAL and HCAL energy deposits.

Jets are clustered from PF candidates using the anti-$k_{{\textrm{T}}}$ algorithm with a distance parameter of 0.4 [22, 23]. The jet momentum is determined as the vectorial sum of all particle momenta in the jet, and is found from simulation to be, on average, within 5 to 10% of the true momentum over the whole transverse momentum ($p_{{\textrm{T}}}$) spectrum and detector acceptance. Additional pp interactions within the same or nearby bunch crossings (pileup) can contribute additional tracks and calorimetric energy depositions, increasing the apparent jet momentum. To mitigate this effect, tracks identified as originating from pileup vertices are discarded and an offset correction is applied to correct for remaining contributions. Jet energy corrections are derived from simulation studies so that the average measured energy of jets becomes identical to that of particle level jets. In situ measurements of the momentum balance in dijet, photon + jet, $\hbox {Z}+$jet, and multijet events are used to determine any residual differences between the jet energy scale in data and in simulation, and appropriate corrections are made [24]. Additional selection criteria are applied to each jet to remove jets potentially dominated by instrumental effects or reconstruction failures. The jet energy resolution amounts typically to 15–20% at 30$\,\text {Ge}\hspace{-.08em}\text {V}$, 10% at 100$\,\text {Ge}\hspace{-.08em}\text {V}$, and 5% at 1 TeV [24]. Jets originating from b quarks are identified using the DeepJet algorithm [17, 25, 26]. This has an efficiency of approximately 78%, at a misidentification probability of 1% for light-quark and gluon jets and 12% for charm-quark jets [17, 26].

The missing transverse momentum vector, ${\vec p}_{{\textrm{T}}}^{\hspace{1.0pt}\text {miss}}$, is computed as the negative vector sum of the transverse momenta of all the PF candidates in an event, and its magnitude is denoted as $p_{{\textrm{T}}} ^\text {miss}$ [27]. The ${\vec p}_{{\textrm{T}}}^{\hspace{1.0pt}\text {miss}}$ is modified to account for corrections to the energy scale of the reconstructed jets in the event.

The momentum resolution for electrons with $p_{{\textrm{T}}} \approx 45\,\text {Ge}\hspace{-.08em}\text {V}$ from $\hbox {Z} \rightarrow \hbox {ee}$ decays ranges from 1.6 to 5.0%. It is generally better in the barrel region than in the endcaps, and also depends on the bremsstrahlung energy emitted by the electron as it traverses the material in front of the ECAL [28, 29].

Muons are measured in the pseudorapidity range $|\eta | < 2.4$, with detection planes made using three technologies: drift tubes, cathode strip chambers, and resistive plate chambers. Matching muons to tracks measured in the silicon tracker results in a relative transverse momentum resolution, for muons with $p_{{\textrm{T}}}$ up to 100$\,\text {Ge}\hspace{-.08em}\text {V}$, of 1% in the barrel and 3% in the endcaps. The $p_{{\textrm{T}}}$ resolution in the barrel is better than 7% for muons with $p_{{\textrm{T}}}$ up to 1 TeV [30].

## Data samples and event selection

The analyzed data sample has been collected with the CMS detector in 2016 at a center-of-mass energy $\sqrt{s} = 13\,\hbox {TeV}$. It corresponds to an integrated luminosity of 36.3$\,\text {fb}^{-1}$ [31]. Events are required to pass a single-electron trigger with a $p_{{\textrm{T}}}$ threshold for isolated electrons of 27$\,\text {Ge}\hspace{-.08em}\text {V}$ or a single-muon trigger with a minimum threshold on the $p_{{\textrm{T}}}$ of an isolated muon of 24$\,\text {Ge}\hspace{-.08em}\text {V}$ [32].

Simulated $\hbox {t}\bar{\hbox {t}}$ signal events are generated with the powheg  v2 ME generator [33–35], pythia8.219 PS [36], and use the CP5 underlying event tune [16] with top quark mass values, $m_{\textrm{t}}^\text {gen}$, of 169.5, 172.5, and 175.5$\,\text {Ge}\hspace{-.08em}\text {V}$. To model parton distribution functions (PDFs), the NNPDF3.1 next-to-next-to-leading order (NNLO) set [37, 38] is used with the strong coupling constant set to $\alpha _\textrm{S} = 0.118$. The various background samples are simulated with the same ME generators and matching techniques [39–43] as in Ref. [13]. The background processes are $\hbox {W}/\hbox {Z}$ + jets, single-top, diboson, and events composed uniquely of jets produced through the strong interaction, referred to as quantum chromodynamics (QCD) multijet events. The PS simulation and hadronization is performed with pythia8, using the CUETP8M1 tune [44].

All of the simulated samples are processed through a full simulation of the CMS detector based on Geant4 [45] and are normalized to their predicted cross section described in Refs. [46–49]. The effects of pileup are included in the simulation and the pileup distribution in simulation is weighted to match the pileup in the data. The jet energy response and resolution in simulated events are corrected to match the data [24]. In addition, the b-jet identification (b tagging) efficiency and misidentification rate [25], and the lepton trigger and reconstruction efficiencies are corrected in simulation [28, 30].

**Fig. 1:**
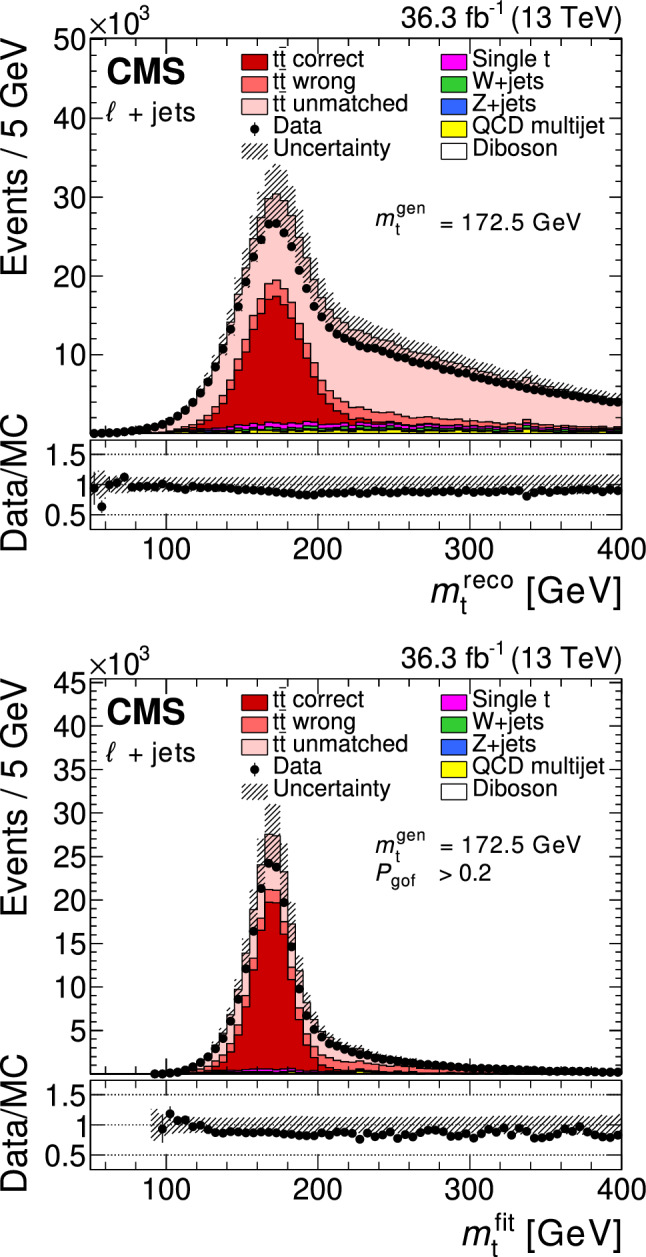
The top quark mass distribution before (upper) and after (lower) the $P_\text {gof} > 0.2$ selection and the kinematic fit. For the simulated $\hbox {t}\bar{\hbox {t}}$ events, the jet-parton assignments are classified as correct, wrong, and unmatched permutations, as described in the text. The uncertainty bands contain statistical uncertainties in the simulation, normalization uncertainties due to luminosity and cross section, jet energy correction uncertainties, and all uncertainties that are evaluated from event-based weights. A large part of the depicted uncertainties on the expected event yields are correlated. The lower panels show the ratio of data to the prediction. A value of $m_{\textrm{t}}^\text {gen} = 172.5 \,\text {Ge}\hspace{-.08em}\text {V}$ is used in the simulation

Events are selected with exactly one isolated electron (muon) with $p_{{\textrm{T}}} > 29$ (26)$\,\text {Ge}\hspace{-.08em}\text {V}$ and $|\eta |< 2.4$ that is separated from PF jet candidates with $\varDelta R=\sqrt{\smash [b]{{(\varDelta \eta )}^2+{(\varDelta \phi )}^2}} >0.3$ (0.4), where $\varDelta \eta$ and $\varDelta \phi$ are the differences in pseudorapidity and azimuth (in radians) between the jet and lepton candidate. The four leading jet candidates in each event are required to have $p_{{\textrm{T}}} >30\,\text {Ge}\hspace{-.08em}\text {V}$ and $|\eta |<2.4$. Only these four jets are used in further reconstruction. Exactly two b-tagged jets are required among the four selected jets, yielding 287 842 (451 618) candidate events in the electron + jets (muon + jets) decay channel.

To check the compatibility of an event with the $\hbox {t}\bar{\hbox {t}}$ hypothesis, and to improve the resolution of the reconstructed quantities, a kinematic fit [50] is performed. For each event, the inputs to the algorithm are the momenta of the lepton and of the four leading jets, ${\vec p}_{{\textrm{T}}}^{\hspace{1.0pt}\text {miss}}$, and the resolutions of these variables. The fit constrains these quantities to the hypothesis that two heavy particles of equal mass are produced, each one decaying to a b quark and a W boson, with the invariant mass of the latter constrained to 80.4$\,\text {Ge}\hspace{-.08em}\text {V}$. The kinematic fit then minimizes $\chi ^{2} \equiv ({\textbf{x}}-{\textbf{x}}^{m})^{\textrm{T}}G({\textbf{x}}-{\textbf{x}}^{m})$ where ${\textbf{x}}^{m}$ and ${\textbf{x}}$ are the vectors of the measured and fitted momenta, respectively, and *G* is the inverse covariance matrix, which is constructed from the uncertainties in the measured momenta. The masses are fixed to 5$\,\text {Ge}\hspace{-.08em}\text {V}$ for the b quark and to zero for the light quarks and leptons. The two b-tagged jets are candidates for the b quark in the $\hbox {t}\bar{\hbox {t}}$ hypothesis, while the two jets that are not b tagged serve as candidates for the light quarks from the hadronically decaying W boson. This leads to two possible parton-jet assignments, each with two solutions for the longitudinal component of the neutrino momentum, and four different permutations per event. For simulated $\hbox {t}\bar{\hbox {t}}$ events, the parton-jet assignments can be classified as correct permutations, wrong permutations, and unmatched permutations, where, in the latter case, at least one quark from the $\hbox {t}\bar{\hbox {t}}$ decay is not unambiguously matched within a distance of $\varDelta R <0.4$ to any of the four selected jets.

The goodness-of-fit probability, $P_\text {gof} = \exp (-\chi ^{2}/2)$, is used to determine the most likely parton-jet assignment. For each event, the observables from the permutation with the highest $P_\text {gof}$ value are the input to the $m_{\textrm{t}}$ measurement. In addition, the events are categorized as either $P_\text {gof} < 0.2$ or $P_\text {gof} > 0.2$, matching the value chosen in Ref. [13]. Requiring $P_\text {gof} > 0.2$ yields 87 265 (140 362) $\hbox {t}\bar{\hbox {t}}$ candidate events in the electron+jets (muon+jets) decay channel and has a predicted signal fraction of 95%. This selection improves the fraction of correctly reconstructed events from 20 to 47%. Figure 1 shows the distribution of the invariant mass of the hadronically decaying top quark candidate before ($m_{\textrm{t}}^\text {reco}$) and after ($m_{\textrm{t}}^\text {fit}$) the $P_\text {gof} > 0.2$ selection and the kinematic fit. A large part of the depicted uncertainties on the expected event yields are correlated. Hence, the overall normalization of the simulation agrees within the uncertainties, although the simulation predicts 10% more events in all distributions. For the final measurement, the simulation is normalized to the number of events observed in data.

## Observables and systematic uncertainties

Different observables are used per event based on its $P_\text {gof}$ value. The cases and observables are listed in Table 1.

**Table 1:** The overall list of different input histograms and their inclusion in a certain histogram set. A histogram marked with “$\times$” is included in a set (measurement)

Histogram		Set label				
Observable	Category	*1D*	*2D*	*3D*	*4D*	*5D*
$m_{\textrm{t}}^\text {fit}$	$P_\text {gof} > 0.2$	$\times$	$\times$	$\times$	$\times$	$\times$
$m_{{\textrm{W}}}^\text {reco}$	$P_\text {gof} > 0.2$		$\times$	$\times$	$\times$	$\times$
$m_{\ell {\textrm{b}}}^\text {reco}$	$P_\text {gof} < 0.2$			$\times$	$\times$	$\times$
$m_{\ell {\textrm{b}}}^\text {reco}/m_{\textrm{t}}^\text {fit}$	$P_\text {gof} > 0.2$				$\times$	$\times$
$R_{{\textrm{bq}}}^\text {reco}$	$P_\text {gof} > 0.2$					$\times$

**Fig. 2:**
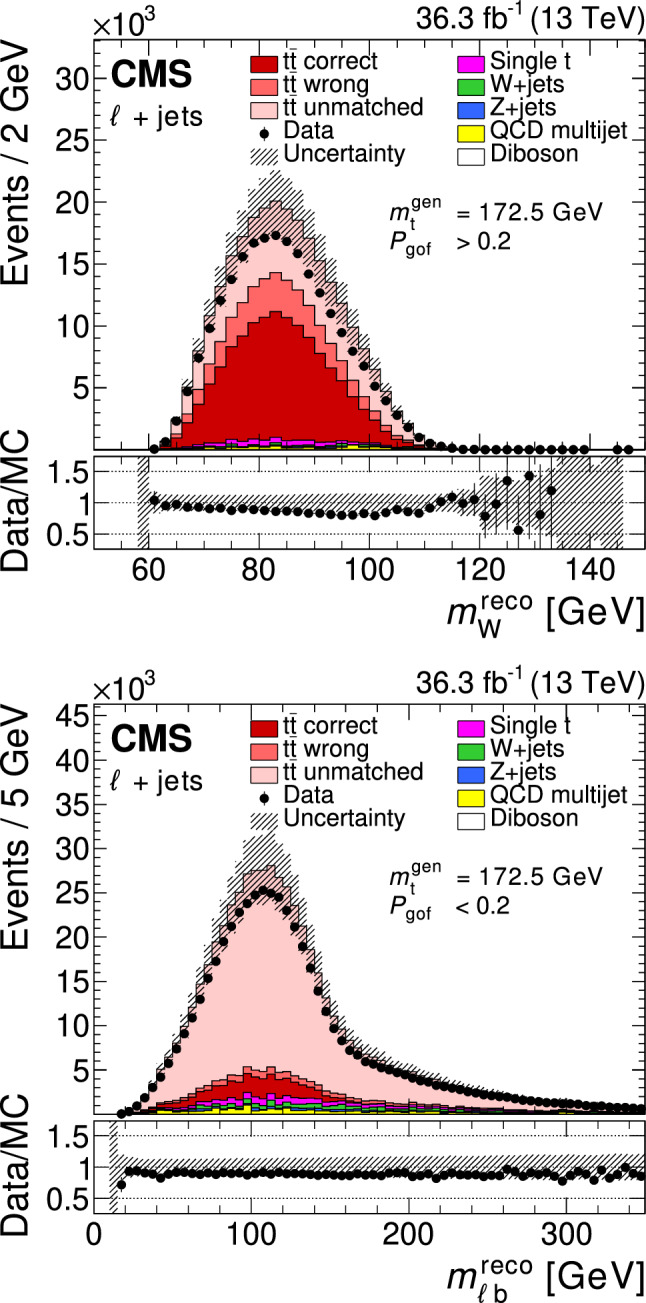
The distributions of the reconstructed W boson mass for the $P_\text {gof} > 0.2$ category (upper) and of the invariant mass of the lepton and the jet assigned to the semileptonic decaying top quark for the $P_\text {gof} < 0.2$ category (lower). The uncertainty bands contain statistical uncertainties in the simulation, normalization uncertainties due to luminosity and cross section, jet energy correction uncertainties, and all uncertainties that are evaluated from event-based weights. A large part of the depicted uncertainties on the expected event yields are correlated. The lower panels show the ratio of data to the prediction. A value of $m_{\textrm{t}}^\text {gen} = 172.5 \,\text {Ge}\hspace{-.08em}\text {V}$ is used in the simulation

For events with $P_\text {gof} > 0.2$, the mass of the top quark candidates from the kinematic fit, $m_{\textrm{t}}^\text {fit}$, shows a very strong dependence on $m_{\textrm{t}}$ and is the main observable in this analysis. Only events with $m_{\textrm{t}}^\text {fit}$ values between 130 and 350 $\,\text {Ge}\hspace{-.08em}\text {V}$ are used in the measurement. The high mass region is included to constrain the contribution of unmatched events to the peak. For events with $P_\text {gof} < 0.2$, the invariant mass of the lepton and the b-tagged jet assigned to the semileptonically decaying top quark, $m_{\ell {\textrm{b}}}^\text {reco}$, is shown in Fig. 2 (lower). For most $\hbox {t}\bar{\hbox {t}}$ events, a low $P_\text {gof}$ value is caused by assigning a wrong jet to the W boson candidate, while the correct b-tagged jets are the candidates for the b quark in 60% of these events. Hence, $m_{\ell {\textrm{b}}}^\text {reco}$ preserves a good $m_{\textrm{t}}$ dependence and adds additional sensitivity to the measurement. The measurement is limited to $m_{\ell {\textrm{b}}}^\text {reco}$ values between 0 and 300$\,\text {Ge}\hspace{-.08em}\text {V}$. While a similar observable has routinely been used in $m_{\textrm{t}}$ measurements in the dilepton channel [51, 52], this is the first application of this observable in the lepton + jets channel.

**Fig. 3:**
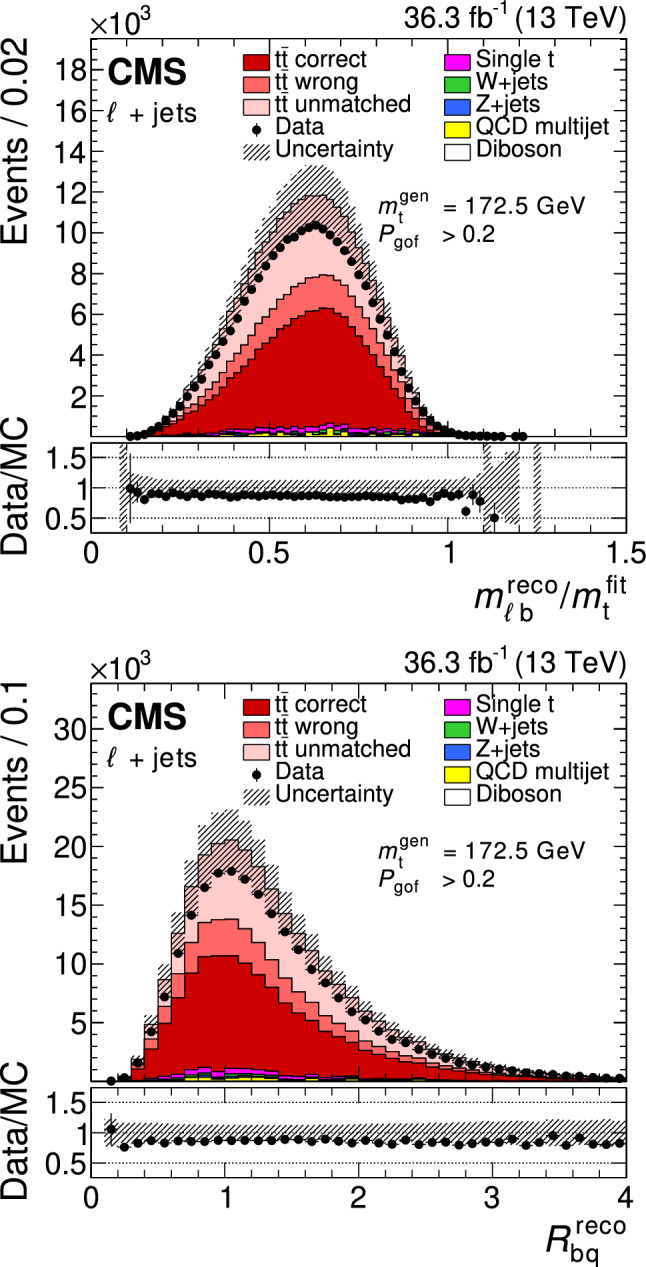
The distributions of $m_{\ell {\textrm{b}}}^\text {reco}/m_{\textrm{t}}^\text {fit}$ (upper) and of $R_{{\textrm{bq}}}^\text {reco}$ (lower), both for the $P_\text {gof} > 0.2$ category. The uncertainty bands contain statistical uncertainties in the simulation, normalization uncertainties due to luminosity and cross section, jet energy correction uncertainties, and all uncertainties that are evaluated from event-based weights. A large part of the depicted uncertainties on the expected event yields are correlated. The lower panels show the ratio of data to the prediction. A value of $m_{\textrm{t}}^\text {gen} = 172.5 \,\text {Ge}\hspace{-.08em}\text {V}$ is used in the simulation

Additional observables are used in parallel for the mass extraction to constrain systematic uncertainties. In previous analyses by the CMS Collaboration in the lepton + jets channel [11, 13], the invariant mass of the two untagged jets before the kinematic fit, $m_{{\textrm{W}}}^\text {reco}$, has been used together with $m_{\textrm{t}}^\text {fit}$, mainly to reduce the uncertainty in the jet energy scale and the jet modeling. Its distribution is shown in Fig. 2 (upper) and the region between 63 and 110$\,\text {Ge}\hspace{-.08em}\text {V}$ is used in the measurement. As $m_{{\textrm{W}}}^\text {reco}$ is only sensitive to the energy scale and modeling of light flavor jets, two additional observables are employed to improve sensitivity to the scale and modeling of jets originating from b quark. These are the ratio $m_{\ell {\textrm{b}}}^\text {reco}/m_{\textrm{t}}^\text {fit}$, and the ratio of the scalar sum of the transverse momenta of the two b-tagged jets ($\hbox {b}1$, $\hbox {b}2$), and the two non-b-tagged jets ($\hbox {q}1$, $\hbox {q}2$), $R_{{\textrm{bq}}}^\text {reco} = (p_{{\textrm{T}}} ^{\hbox {b}1} + p_{{\textrm{T}}} ^{\hbox {b}2})/(p_{{\textrm{T}}} ^{\textrm{q}1}+p_{{\textrm{T}}} ^{\textrm{q}2})$. Their distributions are shown in Fig. 3. While $m_{\textrm{t}}^\text {fit}$ and $m_{{\textrm{W}}}^\text {reco}$ have been used by the CMS Collaboration in previous analyses in the lepton + jets channel, $m_{\ell {\textrm{b}}}^\text {reco}$, $m_{\ell {\textrm{b}}}^\text {reco}/m_{\textrm{t}}^\text {fit}$, and $R_{{\textrm{bq}}}^\text {reco}$ are new additions. However, $R_{{\textrm{bq}}}^\text {reco}$ has been used in the lepton + jets channel by the ATLAS Collaboration [10, 53].

The distributions of the five observables are affected by uncertainties in the modeling and the reconstruction of the simulated events. These sources of systematic uncertainties are nearly identical to those in the previous measurements [13, 54]. The only difference is that we no longer include a systematic uncertainty related to the choice of ME generator as this uncertainty would overlap with the uncertainty we find from varying the default ME generator parameters. The considered sources are summarized in the categories listed below.

- *Method calibration:* In the previous measurements [13, 54], the limited size of the simulated samples for different values of $m_{\textrm{t}}^\text {gen}$ lead to an uncertainty in the calibration of the mass extraction method. In the new profile likelihood approach, the statistical uncertainty in the top quark mass dependence due to the limited sample size is included via nuisance parameters.
- *Jet energy correction* (JEC): Jet energies are scaled up and down according to the $p_{{\textrm{T}}}$- and $\eta$-dependent data/simulation uncertainties [24, 55]. Each of the 25 individual uncertainties in the jet energy corrections is represented by its own nuisance parameter.
- *Jet energy resolution* (JER): Since the JER measured in data is worse than in simulation, the simulation is modified to correct for the difference [24, 55]. The jet energy resolution in the simulation is varied up and down within the uncertainty. The variation is evaluated independently for two $|\eta _{\text {jet}} |$ regions, split at $|\eta _{\text {jet}} |=1.93$.
- *b tagging:* The $p_{{\textrm{T}}}$-dependent uncertainty of the b-tagging efficiencies and misidentification rates of the DeepJet tagger [17, 26] are taken into account by reweighting the simulated events accordingly.
- *Pileup:* To estimate the uncertainty from the determination of the number of pileup events and the reweighting procedure, the inelastic pp cross section [56] used in the determination is varied by $\pm 4.6\%.$
- *Background* (BG): The main uncertainty in the background stems from the uncertainty in the measurements of the cross sections used in the normalization. The normalization of the background samples is varied by ±10% for the single top quark samples [57, 58], ±30% for the $\hbox {W}+$jets samples [59], ±10% for the $\hbox {Z}+$jets [60] and for the diboson samples [61, 62], and ±100% for the QCD multijet samples. The size of the variations is the same as in the previous measurement [13] in this channel. The uncertainty in the luminosity of 1.2% [31] is negligible compared to these variations.
- *Lepton scale factors* (SFs) *and momentum scale:* The simulation-to-data scale factors for the trigger, reconstruction, and selection efficiencies for electrons and muons are varied within their uncertainties. In addition, the lepton energy in simulation is varied up and down within its uncertainty.
- *JEC flavor:* The difference between Lund string fragmentation and cluster fragmentation is evaluated by comparing pythia 6.422 [63] and herwig++  2.4 [64]. The jet energy response is compared separately for each jet flavor [24].
- *b-jet modeling*  (bJES): The uncertainty associated with the fragmentation of b quark is split into four components. The Bowler–Lund fragmentation function is varied symmetrically within its uncertainties, as determined by the ALEPH and DELPHI Collaborations [65, 66]. The difference between the default pythia setting and the center of the variations is included as an additional uncertainty. As an alternative model of the fragmentation into b hadrons, the Peterson fragmentation function is used and the difference obtained relative to the Bowler–Lund fragmentation function is assigned as an uncertainty. The third uncertainty source taken into account is the semileptonic b-hadron branching fraction, which is varied by ${-}0.45$ and ${+}0.77\%$, motivated by measurements of $\hbox {B}^{0}/\hbox {B}^{+}$ decays and their corresponding uncertainties [8] and a comparison of the measured branching ratios to pythia.
- *PDF:* The default PDF set in the simulation, NNPDF3.1 NNLO [37, 38], is replaced with the CT14 NNLO [67] and MMHT 2014 NNLO [68] PDFs via event weights. In addition, the default set is varied with 100 Hessian eigenvectors [38] and the $\alpha _\textrm{S}$ value is changed to 0.117 and 0.119. All described variations are evaluated for their impact on the measurement and the negligible variations are later omitted to reduce the number of nuisance parameters.
- *Renormalization and factorization scales:* The renormalization and factorization scales for the ME calculation are varied independently and simultaneously by factors of 2 and 1/2. This is achieved by reweighting the simulated events. The independent variations were checked and it was found to be sufficient to include only the simultaneous variations as a nuisance parameter.
- *ME to PS matching:* The matching of the powheg ME calculations to the pythia PS is varied by shifting the parameter $h_{\text {damp}}=1.58^{+0.66}_{-0.59}$ [69] within its uncertainty.
- *ISR and FSR:* For initial-state radiation (ISR) and final-state radiation (FSR), 32 decorrelated variations of the renormalization scale and nonsingular terms for each branching type (${\textrm{g}}\rightarrow {\textrm{gg}}$, ${\textrm{g}}\rightarrow \textrm{q}\bar{\textrm{q}}$, $\textrm{q}\rightarrow {\textrm{qg}}$, and ${\textrm{X}} \rightarrow {\textrm{Xg}}$ with ${\textrm{X}}= \hbox {t}\text { or }\hbox {b}$) are applied using event weights [70]. The scale variations correspond to a change of the respective PS scale in pythia by factors of 2 and 1/2. This approach is new compared to the previous analysis [13], which only evaluated correlated changes in the FSR and ISR PS scales.
- *Top quark* $p_{{\textrm{T}}}$: Recent calculations suggest that the top quark $p_{{\textrm{T}}}$ spectrum is strongly affected by NNLO effects [71–73]. The $p_{{\textrm{T}}}$ of the top quark in simulation is varied to match the distribution measured by CMS [74, 75]. The default simulation is not corrected for this effect, but this variation is included via a nuisance parameter in the $m_{\textrm{t}}$ measurement.
- *Underlying event:* Measurements of the underlying event have been used to tune pythia parameters describing nonperturbative QCD effects [16, 44]. The parameters of the tune are varied within their uncertainties.
- *Early resonance decays:* Modeling of color reconnection introduces systematic uncertainties, which are estimated by comparing different CR models and settings. In the default sample, the top quark decay products are not included in the CR process. This setting is compared to the case of including the decay products by enabling early resonance decays in pythia8.
- *CR modeling:* In addition to the default model used in pythia8, two alternative CR models are used, namely a model with string formation beyond leading color (“QCD inspired”) [76] and a model allowing the gluons to be moved to another string (“gluon move”) [77]. Underlying event measurements are used to tune the parameters of all models [78]. For each model, an individual nuisance parameter is introduced.

## Mass extraction method

A maximum likelihood (ML) fit to the selected events is employed to measure $m_{\textrm{t}}$. The evaluated likelihood ratio $\lambda (m_{\textrm{t}}, \vec {\theta }, \vec {\beta }, \varOmega | \text {data})$ depends not only on $m_{\textrm{t}}$, but also on three sets of nuisance parameters. The nuisance parameters, $\vec {\theta }$, incorporate the uncertainty in systematic effects, while the statistical nuisance parameters, $\vec {\beta }$ and $\varOmega$, incorporate the statistical uncertainties in the simulation. The parameters $\vec {\beta }$ account for the limited sample size of the default simulation and the parameters $\varOmega$ account for the limited size in the simulated samples for variations of $m_{\textrm{t}}$ and of the uncertainty sources. All nuisance parameters are normalized such that a value of 0 represents the absence of the systematic effect and the values $\pm 1$ correspond to a variation of the systematic effect by one standard deviation up or down. The RooFit [79] package is used to define and evaluate all the functions. The minimum of the negative log-likelihood $-2 \ln \lambda (m_{\textrm{t}}, \vec {\theta }, \vec {\beta }, \varOmega | \text {data})$ is found with the Minuit2 package [80].

The data are characterized by up to four observables per event, as mentioned in Sect. 4. The events are split into the electron + jets and the muon + jets channels. The input to the ML fit is a set of one-dimensional histograms of the observables, $x_i$, in the two $P_\text {gof}$ categories. For each histogram, a suitable probability density function $P(x_i |m_{\textrm{t}}, \vec {\theta }, \vec {\beta }, \varOmega )$ is derived from simulation.

The probability density function for the $m_{\textrm{t}}^\text {fit}$ histograms is approximated by the sum of a Voigt profile (the convolution of a Cauchy–Lorentz distribution and a Gaussian distribution) for the correctly reconstructed $\hbox {t}\bar{\hbox {t}}$ candidates and Chebyshev polynomials for the remaining events. For all other observables, a binned probability density function is used that returns the relative fraction of events per histogram bin. Here, eight bins are used for each observable and the width of the bins is chosen so that each bin has a similar number of selected events for the default simulation ($m_{\textrm{t}}^\text {gen} = 172.5\,\text {Ge}\hspace{-.08em}\text {V}$). For the following, we denote the parameters of the probability density functions as $\vec {\alpha }$. All the functions $P_i(x_i | \vec {\alpha })$ are normalized to the number of events in the histogram for the observable $x_i$, so only shape information is used in the ML fit. Hence, the parameters $\vec {\alpha }$ are correlated even for the binned probability density function. The dependence of these parameters on $m_{\textrm{t}}$ and $\vec {\theta }$ is assumed to be linear. The full expression is for a component $\alpha _k$ of $\vec {\alpha }$

$$\begin{aligned} \alpha _k= & {} C_k \Bigl (1 + d_k \ \Bigl [ \alpha _k^0 + \sigma ^\alpha _k \ \beta _k + s_k^0 \ (m_{\textrm{t}}-172.5\,\text {Ge}\hspace{-.08em}\text {V}) \\{} & {} + \sigma _k^0 \varOmega _k^0 \Bigr ]\Bigr ) \ \prod _l \Bigl (1+ d_k \ \Bigl [s_k^l \ \theta _l + \sigma _k^l \ \varOmega _k^l \Bigr ] \Bigr ), \end{aligned}$$

with *l* indicating the nuisance parameter. For the nuisance parameters corresponding to the FSR PS scale variations, the linear term, $s_k^l \ \theta _l$, is replaced with a second-order polynomial. With these expressions for the parameters $\alpha _k$, the probability density function, $P_i(x_i | \vec {\alpha })$, for an observable $x_i$ becomes the function $P_i(x_i | m_{\textrm{t}}, \vec {\theta }, \vec {\beta }, \varOmega )$ mentioned above.

The model parameter $\alpha _k^0$ is determined by a fit to the default simulation, while the linear dependencies of $\alpha _k$ on $m_{\textrm{t}}$ or a component $\theta _l$ of $\vec {\theta }$ are expressed with the model parameters $s_k^0$ and $s_k^l$, respectively. The parameter $s_k^0$ is determined from a simultaneous fit to simulated samples, where $m_{\textrm{t}}^\text {gen}$ is varied by ±3$\,\text {Ge}\hspace{-.08em}\text {V}$ from the default value. Along the same lines, the parameters $s_k^l$ are obtained from fits to the simulation of the systematic effect corresponding to the nuisance parameter $\theta _l$. The values of $C_k$ and $d_k$ are chosen ad hoc so that the results of the fits of $\alpha _k^0$, $s_k^0$, and the $s_k^l$ are all of the same order of magnitude and with a similar statistical uncertainty. This improves the numerical stability of the final ML fit.

The limited size of the simulated samples for different $m_{\textrm{t}}^\text {gen}$ values gives rise to a calibration uncertainty in $m_{\textrm{t}}$. Hence, additional statistical nuisance parameters, $\beta _k$ and $\varOmega _k^0$, are introduced that account for the statistical uncertainty in the model parameters $\alpha _k^0$ and $s_k^0$, similar to the Barlow–Beeston approach [81, 82]. They are scaled by $\sigma ^\alpha _k$ and $\sigma _k^0$, which are the standard deviations of $\alpha _k^0$ and $s_k^0$ obtained from the fits to the default simulation or the $m_{\textrm{t}}^\text {gen}$-varied samples, respectively. Hence, a statistical nuisance with value $\varOmega _k^0=\pm 1$ changes the corresponding $\alpha _k$, i.e., the shape or the bin content of an observable, by the statistical uncertainty in the $m_{\textrm{t}}$ dependence evaluated at a shift in $m_{\textrm{t}}$ of 1$\,\text {Ge}\hspace{-.08em}\text {V}$. Similarly, the parameters $s_k^l$ contain random fluctuations if they are determined from simulated samples that are statistically independent to the default simulation and of limited size. These fluctuations can lead to overconstraints on the corresponding nuisance parameter $\theta _l$ and, hence, an underestimation of the systematic uncertainty. The nuisance parameters $\varOmega _k^l$ are added to counter these effects and are scaled by parameters $\sigma _k^l$, which are the standard deviations of the $s_k^l$ parameters from the fits to the corresponding samples for the systematic effect. As in the $m_{\textrm{t}}$ case, a value of $\varOmega _k^l=\pm 1$ changes the corresponding $\alpha _k$ by the statistical uncertainty in the $\theta _l$ dependence evaluated at a shift in $\theta _l$ of 1. Unlike the systematic nuisance parameters $\theta _l$, which affect all $\alpha _k$ collectively, for each $\alpha _k$ there are individual $\varOmega _k^l$ parameters. While this drastically increases the number of fitted parameters in the ML fit to data, this also guarantees that the $\varOmega _k^l$ parameters are hardly constrained by the fit to data and the uncertainty in $m_{\textrm{t}}$ includes the statistical uncertainty in the impact of the systematic effect.

For a single histogram in a set, the products of Poisson probabilities for the prediction $\mu _{i,j} = n_{\text {tot},i}P_i(x_{i,j}|m_{\textrm{t}}, \vec {\theta }, \vec {\beta }, \varOmega )$ and for an alternative model with an adjustable parameter per bin ${{\hat{\mu }}}_{i,j} = n_{i,j}$ are used to compute the likelihood ratio $\lambda _i$ [8], where $x_i$ is the observable, $n_{i,j}$ is the content of bin *j* with bin center $x_{i,j}$, and $n_{\text {tot},i}$ is the total number of entries. Then the combined likelihood ratio for a set with observables $\vec {x}$ is

$$\begin{aligned} \lambda (m_{\textrm{t}}, \vec {\theta }, \vec {\beta }, \varOmega | \text {data})= & {} \left( \prod _i \lambda _i(m_{\textrm{t}}, \vec {\theta }, \vec {\beta }, \varOmega | x_i)\right) \\{} & {} \times \left( \prod _l P(\theta _l)\right) P(\vec {\beta }) P(\varOmega ), \end{aligned}$$

where $P(\theta _l)$, $P(\vec {\beta })$, and $P(\varOmega )$ are the pre-fit probability density functions of the nuisance parameters $\theta _l$, $\vec {\beta }$, and $\varOmega$. The product of the likelihood ratios can be used on the right-hand side of the equation, as all observables are independent in most phase space regions. The probability density functions of the nuisance parameters related to the sources of systematic uncertainties, $P(\theta _l)$, are standard normal distributions. All model parameters $\alpha _k^0$, $s_k^0$, or $s_k^l$ that are related to the same observable and nuisance parameter are obtained together by one fit to the corresponding simulation samples. To take the correlations between these parameters into account, the statistical nuisance parameters $\vec {\beta }$ or $\varOmega$ that incorporate the statistical uncertainty in these parameters are constrained by centered multivariate normal distributions. The covariances of the distributions are set to the correlation matrices obtained by the fits of the corresponding parameters. The latter nuisance parameters and constraints are only included if the model parameters are determined from samples that are statistically independent of the default simulation, like, for example, for the alternative color reconnection models. If the model parameters are determined from samples obtained from varying the default simulation with event-based weights or scaling or smearing of the jet energies, the corresponding $\varOmega$ parameters are fixed to zero and the constraint is removed from $\lambda (m_{\textrm{t}}, \vec {\theta }, \vec {\beta }, \varOmega | \text {data})$.

The mass of the top quark is determined with the profile likelihood fit for different sets of data histograms. The sets and their labels are listed in Table 1.

**Fig. 4:**
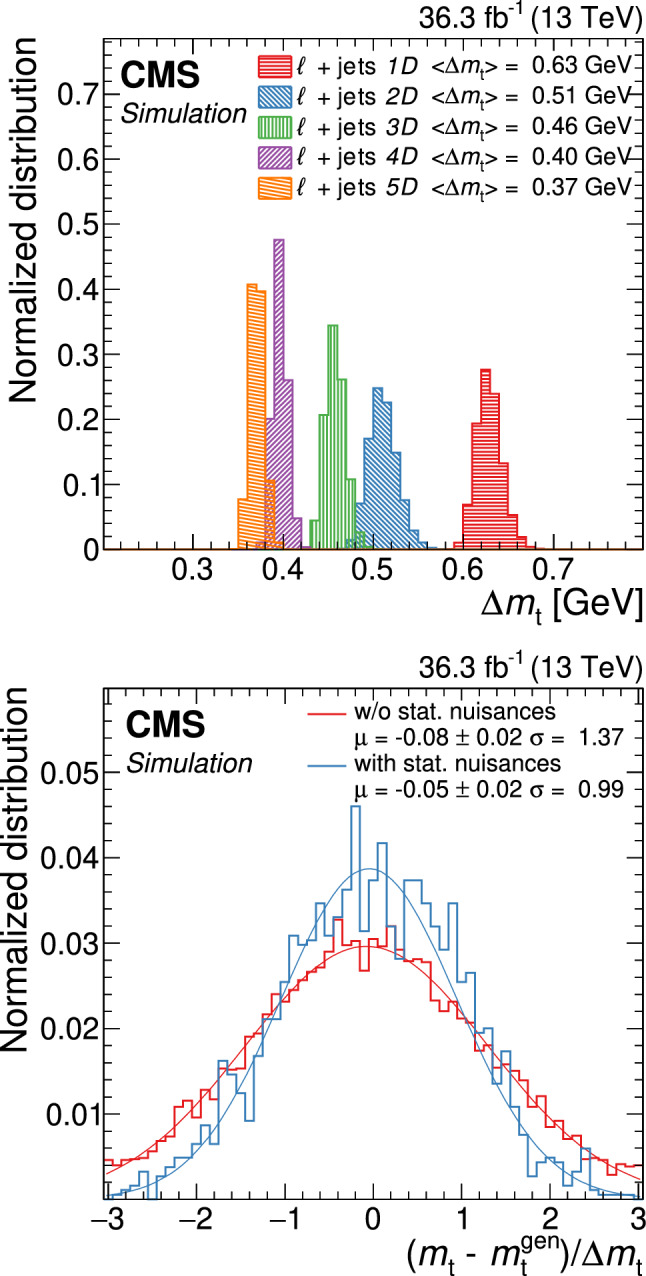
Upper: Comparison of the expected total uncertainty in $m_{\textrm{t}}$ in the combined lepton + jets channel and for the different observable-category sets defined in Table 1. Lower: The difference between the measured and generated $m_{\textrm{t}}$ values, divided by the uncertainty reported by the fit from pseudo-experiments without (red) or with (blue) the statistical nuisance parameters $\vec {\beta }$ and $\varOmega$ in the *5D* ML fit. Also included in the legend are the $\mu$ and $\sigma$ parameters of Gaussian functions (red and blue lines) fit to the histograms

The expected total uncertainty in $m_{\textrm{t}}$ is evaluated for each set defined in Table 1 with pseudo-experiments using the default simulation. The results of the pseudo-experiments are shown in Fig. 4 (upper). The improvements in the data reconstruction and calibration, event selection, simulation, and mass extraction method reduce the uncertainty in the *1D* measurement from 1.09 to 0.63$\,\text {Ge}\hspace{-.08em}\text {V}$, when compared to the previous measurement [13]. The uncertainty in the *2D* measurement improves from 0.63 to 0.50$\,\text {Ge}\hspace{-.08em}\text {V}$. The additional observables and the split into categories further reduce the expected uncertainty down to 0.37$\,\text {Ge}\hspace{-.08em}\text {V}$ for the *5D* set.

The statistical uncertainty is obtained from fits that only have $m_{\textrm{t}}$ as a free parameter. From studies on simulation, it is expected to be 0.07, 0.06, and 0.04$\,\text {Ge}\hspace{-.08em}\text {V}$ in the electron+jets, muon+jets, and the combined (lepton + jets) channels, respectively.

The applied statistical model is verified with additional pseudo-experiments. Here, the data for one pseudo-experiment are generated using probability density functions $P(x_i |m_{\textrm{t}}, \vec {\theta })$ that have the same functional form as the ones used in the ML fit, but their model parameters $\vec {\alpha }$ and all slopes, $s_k^l$ are determined on statistically fluctuated simulations. For the generation of a pseudo-experiment, $m_{\textrm{t}}$ is chosen from a uniform distribution with a mean of 172.5$\,\text {Ge}\hspace{-.08em}\text {V}$ and the same standard deviation as is assumed for the calibration uncertainty. The values of the nuisance parameters $\vec {\theta }$ are drawn from standard normal distributions. The same ML fit that is applied to the collider data is then performed on the pseudo-data. The pseudo-experiments are generated for two cases, specifically, with and without the statistical nuisance parameters $\vec {\beta }$ and $\varOmega$ in the ML fit. Figure 4 (lower) shows the distribution of the differences between the measured and generated $m_{\textrm{t}}$ values, divided by the uncertainty reported by the fit for both cases. A nearly 40% underestimation of the measurement uncertainty can be seen for the case without the statistical nuisance parameters $\vec {\beta }$ and $\varOmega$, while consistency is observed for the method that is employed on data.

In addition, single-parameter fits were performed on pseudo-data sampled from simulation to verify that the mass extraction method is unbiased and reports the correct uncertainty. These tests were done for fits of $m_{\textrm{t}}$ with samples corresponding to mass values of 169.5, 172.5, and 175.5$\,\text {Ge}\hspace{-.08em}\text {V}$, as well as on the simulation of different systematic effects for the fits of the corresponding nuisance parameter.

## Results

**Fig. 5:**
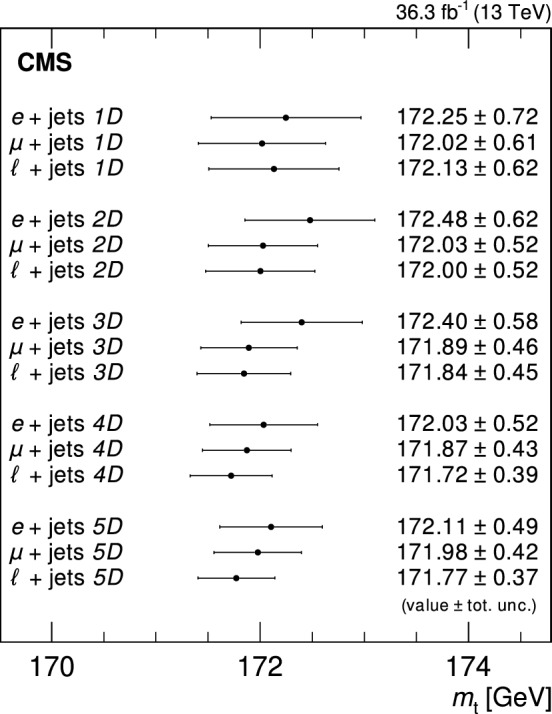
Measurement of $m_{\textrm{t}}$ in the three different channels for the different sets of observables and categories as defined in Table 1

The results of the profile likelihood fits to data are shown in Fig. 5 for the electron + jets, muon + jets, and lepton + jets channels and for the different sets of observables and categories, as defined in Table 1. The observables $m_{{\textrm{W}}}^\text {reco}$, $m_{\ell {\textrm{b}}}^\text {reco}/m_{\textrm{t}}^\text {fit}$, and $R_{{\textrm{bq}}}^\text {reco}$ provide constraints on the modeling of the $\hbox {t}\bar{\hbox {t}}$ decays in addition to the observables $m_{\textrm{t}}^\text {fit}$ and $m_{\ell {\textrm{b}}}^\text {reco} |_{P_\text {gof} <0.2}$, which are highly sensitive to $m_{\textrm{t}}$. With the profile likelihood method, these constraints not only reduce the uncertainty in $m_{\textrm{t}}$, but also change the measured $m_{\textrm{t}}$ value, as they effectively alter the parameters of the reference $\hbox {t}\bar{\hbox {t}}$ simulation. When additional observables are included, the measurement in the lepton + jets channel yields a smaller mass value than the separate channels because of the correlations between the channels.

The *5D* fit to the selected events results in the best precision and yields in the respective channels:

$$\begin{aligned} \text {Electron+jets:}&m_{\textrm{t}} ^{\textit{5D}}&= 172.11\pm 0.49 \,\text {Ge}\hspace{-.08em}\text {V},\\ \text {Muon+jets:}&m_{\textrm{t}} ^{\textit{5D}}&= 171.98\pm 0.42 \,\text {Ge}\hspace{-.08em}\text {V},\\ \text {Lepton+jets:}&m_{\textrm{t}} ^{\textit{5D}}&= 171.77\pm 0.37 \,\text {Ge}\hspace{-.08em}\text {V}. \end{aligned}$$

**Fig. 6:**
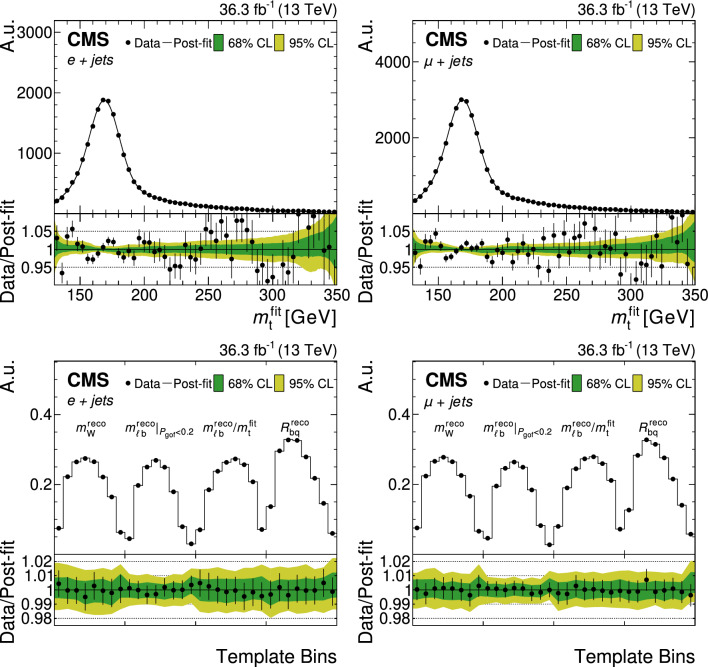
Distribution of $m_{\textrm{t}}^\text {fit}$ (upper) and the additional observables (lower) that are the input to the *5D* ML fit and their post-fit probability density functions for the combined fit to the electron+jets (left) and muon+jets (right) channels. For the additional observables results, the events in each bin are divided by the bin width. The lower panels show the ratio of data and post-fit template values. The green and yellow bands represent the 68 and 95% confidence levels in the fit uncertainty

The comparisons of the data distributions and the post-fit *5D* model are shown in Fig. 6. While the binned probability density functions of the model describe the corresponding observables well, significant deviations between the data and the model can be observed in the peak region of the $m_{\textrm{t}}^\text {fit}$ observable. These deviations are also observed in simulation and stem from the fact that effectively only two parameters, the peak position and its average width, are used in the model to describe the peak. Tests with simulation show no bias on the extracted $m_{\textrm{t}}$ value from these deviations. In fact, the small number of parameters should increase the robustness of the measurement as the model is not sensitive to finer details of the peak shape that might be difficult to simulate correctly.

**Fig. 7:**
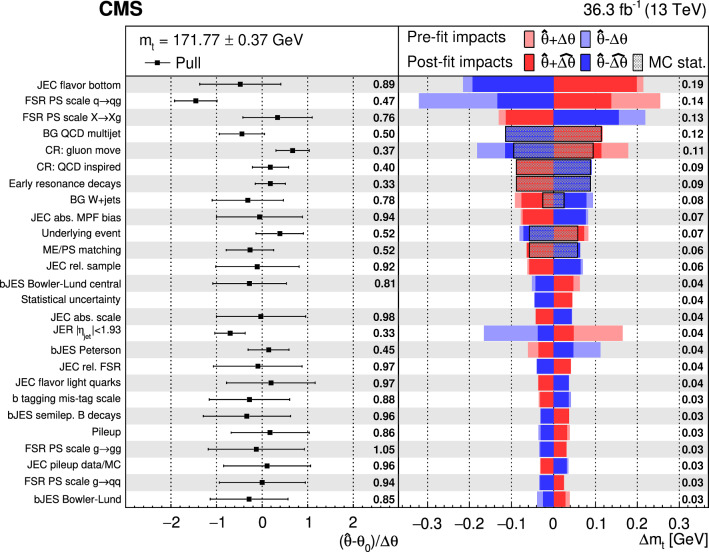
Measurement of $m_{\textrm{t}}$ in the combined lepton + jets channel using the *5D* set of observables and categories. The left plot shows the post-fit pulls on the most important nuisance parameters and the numbers quote the post-fit uncertainty in the nuisance parameter. The right plot shows their pre-fit (lighter colored bars) and post-fit impacts (darker colored bars) on $m_{\textrm{t}}$ for up (red) and down (blue) variations. The post-fit impacts of systematic effects that are affected by the limited size of simulation samples include the contribution from the additional statistical nuisance parameters accounting for the effect. The size of the additional contribution from the statistical nuisance parameters is called MC stat. and shown as gray-dotted areas. The average of the post-fit impacts in $\,\text {Ge}\hspace{-.08em}\text {V}$ for up and down variations is printed on the right. The rows are sorted by the size of the averaged post-fit impact. The statistical uncertainty in $m_{\textrm{t}}$ is depicted in the corresponding row

Figure 7 shows the pulls on the most important systematic nuisance parameters $\theta$ and their impacts on $m_{\textrm{t}}$, $\varDelta m_{\textrm{t}}$, after the fit with the *5D* model. The pulls are defined as $({\hat{\theta }}- \theta _0)/\varDelta \theta$, where ${\hat{\theta }}$ is the measured nuisance parameter value and $\theta _0$ and $\varDelta \theta$ are the mean and standard deviation of the nuisance parameter before the fit. The pre-fit impacts are evaluated by repeating the ML fit with the studied nuisance parameter $\theta$ fixed to ${\hat{\theta }} \pm \varDelta \theta$ and taking the difference in $m_{\textrm{t}}$ between the result of these fits and the measured $m_{\textrm{t}}$. In most cases, the post-fit impacts are evaluated respectively with ${\hat{\theta }}$ and $\widehat{\varDelta \theta }$, where $\widehat{\varDelta \theta }$ is the uncertainty in the nuisance parameter after the fit. However, if the studied systematic nuisance parameter $\theta$ has statistical nuisance parameters $\varOmega$ that account for the statistical uncertainty in the $\theta$-dependence of the model, the combined impact of the systematic and statistical nuisance parameters is plotted in Fig. 7 as post-fit impact. To estimate this combined impact, the likelihood fit is repeated with the corresponding systematic and statistical nuisance parameters fixed to their post-fit values and the quadratic difference of the overall $m_{\textrm{t}}$ uncertainty compared to the default fit is taken. The quadratic difference between the combined impact and the post-fit impact of only the systematic nuisance parameter is interpreted as the effect of the limited size of the systematic simulation samples.

Most nuisance parameters are consistent with their pre-fit values. The largest effect on the measured mass value corresponds to the FSR scale of the ${\textrm{q}}\rightarrow {\textrm{qg}}$ branching type. The effect is caused by the difference in the peak position of $m_{{\textrm{W}}}^\text {reco}$ seen in Fig. 2 (upper). The previous measurements in this channel by the CMS Collaboration assumed correlated FSR PS scales with the same scale choice for jets induced by light quarks and b quark [11, 13]. In that case, a lower peak position in the $m_{{\textrm{W}}}^\text {reco}$ distribution would also cause the $m_{\textrm{t}}^\text {fit}$ peak position to be lower than expected from simulation for a given $m_{\textrm{t}}$ value, resulting in a higher top quark mass value to be measured. In fact, a *5D* fit to data assuming fully correlated FSR PS scale choices yields $m_{\textrm{t}} = 172.20 \pm 0.31\,\text {Ge}\hspace{-.08em}\text {V}$. This value is very close to the previous measurement on the same data of $m_{\textrm{t}} = 172.25 \pm 0.63\,\text {Ge}\hspace{-.08em}\text {V}$ [13].

**Fig. 8:**
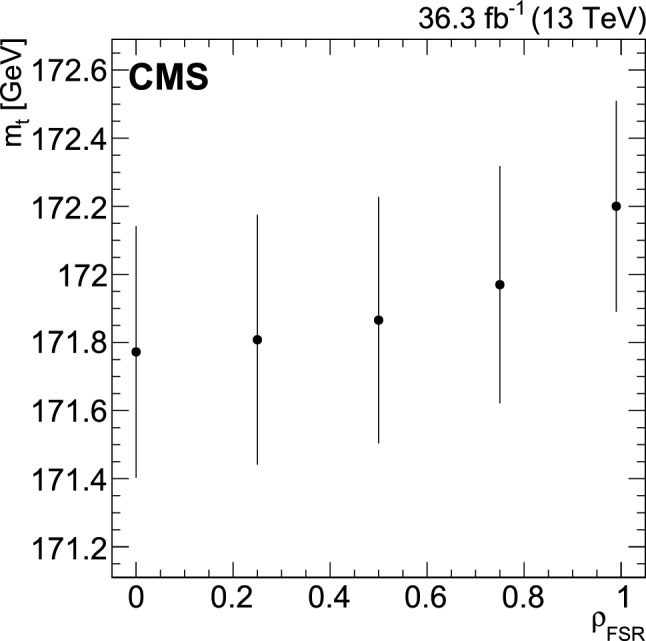
Dependence of the *5D* result on the assumed correlation $\rho _{\text {{FSR}}}$ between the FSR PS scales in the lepton + jets channel

The measurement is repeated for different correlation coefficients ($\rho _{\text {{FSR}}}$) in the pre-fit covariance matrix between the FSR PS scales for the different branching types. The result of this study is shown in Fig. 8. The final result strongly depends on the choice of the correlation coefficient between the FSR PS scales because of the significant deviation for the FSR PS scale of the ${\textrm{q}}\rightarrow {\textrm{qg}}$ branching from the default simulation. However, the assumption of strongly correlated FSR PS scale choices would also significantly reduce the overall uncertainty, as the impacts from the scale choice for gluon radiation from b quark ($\hbox {X}\rightarrow {\textrm{Xg}}$) and light quarks (${\textrm{q}}\rightarrow {\textrm{qg}}$) partially cancel. In addition, there is a tension between the measured nuisance parameter values for the different FSR PS scales, which disfavors a strong correlation. As there is only a small dependence on FSR PS scale correlations at low correlation coefficients ($\rho _{\text {{FSR}}} < 0.5$), and uncorrelated nuisance parameters for the FSR PS scales receive the least constraint from the fit to data, we assume uncorrelated FSR PS scales for this measurement.

Most of the other nuisance parameters that show a strong post-fit constraint correspond to systematic uncertainties that are evaluated on independent samples of limited size. The small sample sizes are expected to bias these nuisances parameter and lead to too small uncertainties. Hence, the nuisance parameters are accompanied by additional statistical nuisance parameters. A comparison of the pre-fit and post-fit impacts where the post-fit impacts include the impact of these statistical nuisance parameters shows that there is an only minimal constraint by the fit on the corresponding systematic uncertainties.

The largest constraint of a nuisance parameter without additional statistical nuisance parameters corresponds to the JER uncertainty. This is expected, as the energy resolution of jets from $\hbox {t}\bar{\hbox {t}}$ decays can be measured much better from the width of the $m_{{\textrm{W}}}^\text {reco}$ distribution than by the extrapolation of the resolution measurement with dijet topologies at much higher transverse momenta [24].

**Table 2:** Comparison of the uncertainty in the top quark mass in the previous measurement [13, 54] and the new *2D* and *5D* results in the lepton + jets channel

	$\delta$ $m_{\textrm{t}}$ [GeV]		
	Previous *2D*	*2D*	*5D*
*Experimental uncertainties*			
Method calibration	0.05	0.02	0.02
JEC	0.18	0.32	0.16
– Intercalibration	0.04	0.10	0.04
– MPFInSitu	0.07	0.15	0.07
– Uncorrelated	0.16	0.21	0.10
Jet energy resolution	0.12	0.12	0.05
b tagging	0.03	0.01	0.03
Lepton SFs and mom. scale		0.00	0.03
Pileup	0.05	0.00	0.03
Background	0.02	0.12	0.15
*Modeling uncertainties*			
JEC flavor	0.39	0.30	0.20
b-jet modeling	0.12	0.15	0.11
PDF	0.02	0.00	0.01
Renorm. and fact. scales	0.01	0.03	0.02
ME/PS matching	0.07	0.06	0.07
ME generator	0.20	–	–
ISR PS scale	0.07	0.01	0.01
FSR PS scale	0.13	0.37	0.21
Top quark $p_{{\textrm{T}}}$	0.01	0.06	0.00
Underlying event	0.07	0.09	0.04
Early resonance decays	0.07	0.13	0.09
CR modeling	0.31	0.15	0.15
*Statistical*	0.08	0.05	0.04
*Total*	0.63	0.52	0.37

Table 2 compares the measurements by the *2D* and *5D* methods with the previous result [13, 54] for the same data-taking period. The JEC uncertainties are grouped following the recommendations documented in Ref. [83]. The uncertainty in $m_{\textrm{t}}$ for one source (row) in this table is evaluated from the covariance matrix of the ML fit by taking the square root of $\text {cov}(m_{\textrm{t}},X) \text {cov}(X,X)^{-1} \text {cov}(X,m_{\textrm{t}})$, where $\text {cov}(m_{\textrm{t}},X)$, $\text {cov}(X,X)$, $\text {cov}(X,m_{\textrm{t}})$ are the parts of the covariance matrix related to $m_{\textrm{t}}$ or the set of nuisance parameters *X* contributing to the source, respectively. The statistical and calibration uncertainties are obtained differently by computing the partial covariance matrix on $m_{\textrm{t}}$ where all other nuisance parameters are removed. The quadratic sum of all computed systematic uncertainties is larger than the uncertainty in $m_{\textrm{t}}$ from the ML fit, as the sum ignores the post-fit correlations between the systematic uncertainty sources.

The *5D* method is the only method that surpasses the strong reduction in the uncertainty in the JEC achieved by the previous analysis that determined $m_{\textrm{t}}$ and in situ an overall jet energy scale factor (JSF). However, the measurement presented here also constrains the jet energy resolution uncertainty that was unaffected by the JSF. The new observables and additional events with a low $P_\text {gof}$ reduce most modeling uncertainties, but lead to a slight increase in some experimental uncertainties. While the usage of weights for the PS variations removes the previously significant statistical component in the PS uncertainties, the introduction of separate PS scales leads to a large increase in the uncertainty in the FSR PS scale, despite the tight constraint on the corresponding nuisance parameters shown in Fig. 7.

The result presented here achieves a considerable improvement compared to all previously published top quark mass measurements. Hence, it supersedes the previously published measurement in this channel on the same data set [13]. The analysis shows the precision that is achievable from direct measurements of the top quark mass. As the uncertainty in the relationship of the direct measurement from simulation templates to a theoretically well-defined top quark mass is currently of similar size, the measurement should fuel further theoretical studies on the topic.

## Summary

The mass of the top quark is measured using LHC proton–proton collision data collected in 2016 with the CMS detector at $\sqrt{s}=13\,\text {Te}\hspace{-.08em}\text {V}$, corresponding to an integrated luminosity of 36.3$\,\text {fb}^{-1}$. The measurement uses a sample of $\hbox {t}\bar{\hbox {t}}$ events containing one isolated electron or muon and at least four jets in the final state. For each event, the mass is reconstructed from a kinematic fit of the decay products to a $\hbox {t}\bar{\hbox {t}}$ hypothesis. A likelihood method is applied using up to four observables per event to extract the top quark mass and constrain the influences of systematic effects, which are included as nuisance parameters in the likelihood. The top quark mass is measured to be $171.77\pm 0.37\,\text {Ge}\hspace{-.08em}\text {V}$. This result achieves a considerable improvement compared to all previously published top quark mass measurements and supersedes the previously published measurement in this channel on the same data set.

## Data Availability

This manuscript has no associated data or the data will not be deposited. [Authors’ comment: Release and preservation of data used by the CMS Collaboration as the basis for publications is guided by the CMS policy as stated in “https://cms-docdb.cern.ch/cgi-bin/PublicDocDB/RetrieveFile?docid=6032 &filename=CMSDataPolicyV1.2.pdf &version=2 CMS data preservation, re-use and open access policy”].
